# Hsa_circ_0003998 promotes epithelial to mesenchymal transition of hepatocellular carcinoma by sponging miR-143-3p and PCBP1

**DOI:** 10.1186/s13046-020-01576-0

**Published:** 2020-06-17

**Authors:** Li-na Song, Guang-lei Qiao, Jian Yu, Chun-mei Yang, Ying Chen, Zhou-feng Deng, Li-hua Song, Li-jun Ma, Hong-li Yan

**Affiliations:** 1 Department of Oncology, Tongren Hospital, Shanghai Jiao Tong University School of Medicine, 1111, Xianxia Road, Shanghai, 200336 China; 2 Third Department of Hepatic Surgery, Eastern Hepatobiliary Surgery Hospital, Naval military Medical University, Shanghai, China; 3 School of Agriculture and Biology, Shanghai Jiao Tong University, 800, Dongchuan road, Shanghai, 201109 China; 4 Department of Laboratory Diagnosis and Reproductive Medical Center, Changhai Hospital, Naval military Medical University, 168, Changhai Road, Shanghai, 200433 China

**Keywords:** Hepatocellular carcinoma, EMT, hsa_circ_0003998, microRNA-143-3p, FOSL2, PCBP1

## Abstract

**Background:**

Circular RNAs (circRNAs) play a critical regulatory role in cancer progression. However, the underlying mechanisms of circRNAs in hepatocellular carcinoma (HCC) metastasis remain mostly unknown.

**Methods:**

Has_circ_0003998 (circ0003998) was identified by RNAs sequencing in HCC patients with /without portal vein tumor thrombus (PVTT) metastasis. The expression level of circ0003998 was further detected by in situ hybridization on tissues microarray (ISH-TMA) and qRT-PCR in 25 HCC patients with PVTT metastasis. Moreover, the 25 HCC patients with PVTT metastasis and 50 HCC patients without PVTT metastasis were recruited together to analyze the correlation between circ0003998 expression and HCC clinical characteristics. Transwell, migration and CCK8 assays, as well as nude mice model of lung or liver metastasis were used to evaluate the role of circ0003998 in epithelial to mesenchymal transition (EMT) in HCC. The regulatory mechanisms of circ0003998 in miR-143-3p and PCBP1 were determined by dual-luciferase reporter assay, nuclear-cytoplasmic fractionation, fluorescent in situ hybridization, RNA pull- down, microRNA sequence, western blot and RNA immunoprecipitation.

**Results:**

Compared with adjacent normal liver tissues (ANL), circ0003998 expression was significantly upregulated in PVTT tissues and HCC tissues, and its expression correlates with the aggressive characteristics of HCC patients. Further assays suggested that circ0003998 promoted EMT of HCC both in vitro and in vivo. Mechanistically, our data indicated that circ0003998 may act as a ceRNA (competing endogenous RNA) of microRNA-143-3p to relieve the repressive effect on EMT-related stimulator, FOSL2; meanwhile, circ0003998 could bind with PCBP1-poly(rC) binding protein 1 (PCBP1) to increase the expression level of EMT-related genes, CD44v6.

**Conclusion:**

Circ0003998 promotes EMT of HCC by circ0003998/miR-143-3p/FOSL2 axis and circ0003998 /PCBP1/CD44v6 axis.

## Background

Hepatocellular carcinoma (HCC) is one of the most common and lethal tumors worldwide due to metastases and recurrence [1, 2]. Epithelial to mesenchymal transition (EMT) is a complex biological process that plays key role in tumor metastases, and it disrupts the intercellular junctions, polarity, order, and consistency of the cells, leading to tumor recurrence [3]. More and more studies have elucidated the indispensable role of EMT in metastatic dissemination of HCC; and it highlighted the need for further study of EMT-related molecular mechanisms in HCC [3, 4].

Covalently closed circular RNAs (circRNAs) are connected by the back-splicing of exons (3′ and 5′ ends) or introns of precursor mRNAs [5]. Recent studies have shown the critical functions of circRNAs, such as microRNAs (miRNAs) or RNA-binding proteins (RBPs) sponging, proteins translation, transcription, and splicing modulation [6, 7]. As reported, circRNAs perform better as diagnostic and therapeutic targets than linear transcripts by virtue of its structural stability, species conservation, and cell/tissue-specificity [8, 9]. More importantly, abnormal expression of non-coding RNAs is closely related to EMT in the metastatic process of cancers [10, 11].

In the present study, we investigated the expression profile of circRNAs in HCC patients with or without portal vein tumor thrombus (PVTT) metastasis using RNA-sequencing (RNA-seq). And we characterized a novel circRNAs, circ0003998 (circBase ID: hsa_circ_0003998) that derived from ADP ribosylation factor guanine nucleotide exchange factor 2 (ARFGEF2) and located at chr20:47570092–47,580,435. We further showed that circ0003998 plays a key role in HCC metastasis through circ0003998/miR-143-3p/FOSL2 axis and circ0003998 /PCBP1/CD44v6 axis.

## Materials and methods

### Patient tissues and cell lines

The study recruited 25 HCC patients with PVTT metastasis (cohort 1) and 50 HCC patients without PVTT metastasis (cohort 2) from the Eastern Hepatobiliary Surgery Hospital (Shanghai, China). The patients with a history of preoperative chemoradiotherapy were excluded. HCC tissues, PVTT tissues, and corresponding adjacent normal liver tissues (ANL) tissues were collected from cohort 1; and another HCC tissues and corresponding ANL tissues were collected from cohort 2. The tissues were verified by two pathologists independently. The study complied with the Declaration of Helsinki and was approved by the Human Ethics Committee of the Eastern Hepatobiliary Surgery Hospital (Shanghai, China). Moreover, all patients in this study provided written informed consent. HCC cell lines (HepG2, HuH7, Hep3B, and PLC/PRF/5) were purchased from the American Type Culture Collection (ATCC, USA). The MHCC97H cells, hepatocyte cell line L02, and HEK293T cells were obtained from the Cell Bank of the Chinese Academy of Sciences (Shanghai, China). No cell lines were contaminated by other cells, such as Hela, as shown by short tandem repeat (STR) data (Biowing Applied Biotechnology Co., Ltd., Shanghai, China) (Additional file 1). All cells were maintained in humidified incubators (37 °C) with 5% CO_2_ and were cultured in Dulbecco’s modified Eagle’s medium (SIGMA, USA) with 10% fetal bovine serum (GIBCO, BRL).

### Total RNA isolation, RNA-seq, and RNase R treatment

Total RNA were isolated from the cultured cells and fresh tissues by Trizol reagent (Invitrogen, USA). Total RNA was extracted by RNAprep pure Tissue Kit (TIANGEN), and rRNA was depleted by GeneRead rRNA Depletion Kit (QIAGEN). The mRNA was purified from total RNA by oligo (dT) magnetic beads, and fragmented into 200–500 bp; the cleaved RNA fragments were reverse-transcribed into cDNA, and enriched by PCR to create the final complementary DNA (cDNA) libraries. The harvested target bands were quantified by Agilent 2100 and then subjected to deep sequencing with the Illumina HiSeq 2000. HCC cells were mixed with RNase R (3 U/μg, Epicentre, Madison, WI) at 37 °C for 15 min, and then qRT-PCR was used to assess the expression stability of circ0003998 as compared to ARFGEF2 mRNA.

### Reverse transcription reaction and quantitative real-time PCR

The first-strand cDNA was synthesized by the PrimeScript RT reagent kit (Takara Bio Inc., China), and the reverse transcription of miRNAs was generated by miRNAs First Strand cDNA Synthesis Poly A Tailing Kit (Sangon Bio Inc., China). SYBR Premix Ex TaqII (Takara Bio Inc., China) was used in Quantitative real-time polymerase chain reaction (qRT-PCR). β-actin and U6 were used as endogenous references for mRNA and miRNAs respectively. The comparative 2^-ΔΔCt^ method was used to calculate the relative fold-change of target expression. The sequences of the primers in this study were showed in Additional file 2. All primers were designed and purchased from Sangon (Shanghai, China).

### Tissue microarray (TMA) and in situ hybridization (ISH)

After dewaxed in xylene and rehydrated with gradient alcohol, the TMAs were digested by protease K (20 μg/mL) at 37 °C. 3% methanol-H2O2 was used to block endogenous peroxidase. Next, TMAs were hybridized with specific digoxin-labeled circ0003998 probe (8 ng/μL, Digoxin- 5′-Digoxin-GGCCTCCTGCAACTTTAATGGCAGATG-Digoxin-3′, Service Biotechnology, Wu han, China) at 37 °C overnight. TMAs were incubated in BSA serum at room temperature for 30 min and then incubated with mouse anti-digoxin labeled peroxidase (anti-DIG-HRP) at 37 °C for 40 min. The TMAs were stained with freshly prepared diaminoaniline (DAB) solution, and the nucleus was stained with Harris hematoxylin stain.

### Fluorescence in situ hybridization (FISH)

FISH assay was used to find the intracellular location of circ0003998. Probes of FISH assay for circ0003998, human U6, and human18S were synthesized by RiboBio Co., Ltd. (Guangzhou, China). Briefly, cells were rinsed with phosphate buffer saline (PBS) and fixed in 4% formaldehyde solution for 10 min at room temperature, and then incubated with 0.5% Triton X-100 solution for 5 min at 4 °C. After pre-hybridization for 30 min, the cells were hybridized with fluorescence-hybridization probes overnight in the dark at 37 °C. Then, cells were washed three times in 4X/2X/1X SSC solution in the dark at 42 °C for 5 min respectively, and laser scanning confocal microscopy was used to visualize the images at 400 × magnification.

### Nuclear-cytoplasmic fractionation

Cytoplasmic and nuclear RNA isolation was detected by the PARIS™ Kit (Invitrogen, USA). Briefly, after washed in PBS, the cultured cells were resuspended in cold cell fraction buffer and then incubated on ice for 10 min. The cells samples were centrifuged at 4 °C to separate the supernatant containing cytoplasmic fractions from the pellet containing nuclear fraction. The nuclear pellet was rewashed by the cell fraction buffer and was lysed by the disruption buffer. Both the cytoplasmic fraction and the nuclear fraction were respectively mixed with 2X lysis/ binding solution, followed by 100% ethanol. After centrifugation and washing, the RNA of nuclear and cytoplasmic were respectively obtained with the eluting solution.

### Establishment of stable cell lines over expressing hsa_circ_0003998

The circ0003998 expressing lentivirus vector (HBLV-circ0003998-GFP-PURO, termed as OE-circ-vector) and its control vector (HBLV-GFP-PURO, termed as NC-circ-vector) were supplied by Hanbio (Shanghai, China). HCC cells were infected with the lentivirus vectorfoloowed by selection with 2 μg/mL puromycin for 1 week.

### Construction of siRNA, plasmids, miRNA mimic, and transient transfection

The small interfering RNAs (siRNAs) of circ0003998 (si-circ0003998) and the corresponding negative control (siRNA-NC) were synthesized by Hanbio (Shanghai, China). The PCBP1 expressing plasmid (OE-PCBP1), FOSL2 expressing plasmid (OE-FOSL2) and the corresponding negative plasmid vector (vector) were provided by Hanbio (Shanghai, China). The siRNAs of PCBP1 (si-PCBP1), siRNAs of FOSL2 (si-FOSL2) were synthesized by GenePharma (Shanghai, China). The microRNA-143-3p mimics (miR-143-3p) and corresponding negative control (miR-143-3p-NC) were synthesized by GenePharma (Suzhou, China). After digestion with Xho I/Xba I, circ0003998 cDNA (full-length: 304 bp) from HepG2 was amplified by qRT-PCR which cloned into pmirGLO Dual-Luciferase miRNA Target Expression Vector (Promega Corporation, USA) to synthesize wild type-plasmid for the luciferase reporter assay (Luci-circ0003998-WT). The corresponding mutant type-plasmid (Luci-circ000 3998-MT) contained with mutated miRNA binding sites and was synthesized by Genscript (Nanjing, China). Lipofectamine 3000 kit (Invitrogen) was used in transient transfection according to the manufacturer’s instructions.

### Wound-healing assay

The cells were seeded in six-well plates and the center of each well was straightly scratched using a 10-μL plastic pipette tip. After floating cells were gently removed with PBS, cells in the six well plates continued to be cultured for 48 h. The wound-healing process were monitored by inverted light microscope.

### Cell counting Kit-8 assay

Cell proliferation was assessed by Cell Counting Kit-8 kit (CCK-8, Dojindo Chemical Laboratory, Kumamoto, Japan). HCC cells (3 × 10^3^/well) were seeded in 96-well plates with 6 replicates. 10 μL/well of CCK8 solution and 90 μL/well of fresh medium were mixed and added to each well at 0, 24, 48, 72, and 96 h, respectively, After incubation for 1.5 h in cell incubator, the absorbance of the medium at 450 nm was measured.

### Migration assay and invasion assay

24-well transwell migration champers (8 μm size, Corning-3422, USA) and pre-biocoated matrigel transwell invasion chambers (8 μm size, Corning-354,480, USA) were used for migration assay and invasion assay respectively. Briefly, a total of 5 × 10^4^ HuH7, 8 × 10^4^ MHCC97H, and 8 × 10^4^ HepG2 cells were resuspended in 200 μL serum-free DMEM medium and were seeded into the inner chambers, respectively. 600 μL DMEM medium containing 10% FBS as the attractant was loaded to the bottom chambers. For migration assay, the cells were incubated for 24 h, and for invasion assay, the cells were incubated for 48 h. After incubation, the cells that migrated or invaded through the pores were fixed with 4% paraformaldehyde, and stained with 0.1% crystal violet, and then counted at least in five random fields under 200 × microscope.

### Luciferase reporter assay

HCC cells or HEK293T cells (3 × 10^5^) were seeded in a 24-well plate overnight. 250 ng of Luci- 0003998-WT/Luci-circ0003998-MT were co-transfected into the cells with 100 nM miR-143-3p mimic/miR-143-3p mimic-NC using Lipofectamine 3000. In 48 h after transfection, firefly and Renilla luciferase fluorescence were detected by the Dual-Luciferase Reporter Assay kit (Promega, USA). The relative luciferase activities were quantified by Firefly/Renilla fluorescence.

### RNA immunoprecipitation (RIP) assay

Magna RIP RNA Binding Protein Immunoprecipitation Kit (Millipore, Billerica, MA, USA) was used in RIP assay according to the manufacturer’s protocol. HepG2-OE cells (2 × 10^7^) were lysed in RIP lysis buffer. 5 μg of human anti-targeted protein antibody or negative anti-IgG antibodies was incubated with magnetic beads for 2 h. Then, 100 μL RIP lysate was incubated with bead-antibody complex in 900 μL RIP Immunoprecipitation buffer (shaking at 4 °C overnight). After the beads were incubated with proteinase K buffer for 30 min at 55 °C, the immunoprecipitated RNAs were finally extracted to further detect the expression levels of circ0003998 by qRT-PCR.

### RNA pull-down

HepG2 cells (2 × 10^7^) were washed in cold PBS and then lysed in a lysis buffer. Biotin-labeled circ0003998 probe (5′-Biotin-aaaTTCCAGTTCTCTGGCCTCCTGCAACTTTAATGGCAGATG TGACTACAT-3′) and control probe (5′-Biotin-aaaATGTAGTCACATCTGCCATTAAAGTTGC AGGAGGCCAGAGAACTGGAA-3′) were synthesized by CloudSeq Biotech Inc. (Shanghai, China). Biotin-coupled probes were bounded on magnetic beads and then incubated with lysates of HepG2-OE cells overnight at 4 °C. After purification, the pull-down product was analyzed by miRNA sequencing and protein spectrum.

### In vivo tumorigenesis and metastasis assays

The animal experiments met the demands of laboratory animal welfare and ethics, and approved by the Institutional Animal Care and Use Committee of Naval Medical University (Shanghai, China). The nude male BALB/c mice (four weeks old) were purchased from Animal Center of Naval Medical University and housed under pathogen-free conditions with standard pellet diet and water. Twenty mice were randomly divided into four groups for the construction of two model. MHCC97H-OE cells (3 × 10^6^) or MHCC97H-NC cells (3 × 10^6^) were injected into the tail vein to establish the lung metastatic model. MHCC97H-OE cells (5 × 10^6^) or MHCC97H-NC cells (5 × 10^6^) were injected into the spleen to establish liver metastatic model. IVIS@ LuminaII system was used to measure the fluorescence value of GFP at excitation wave-length of 488 nm, which monitor the metastases of two types of mice models. Mice were sacrificed with CO_2_; their lungs and livers were stained with H&E and re-examined microscopically for the development of metastatic foci.

### Western blot

HCC cells were lysed in a RIPA buffer (Beyotime Biotechnology, Nantong, China). BCA Protein Assay kit (Beyotime Biotechnology, Nantong, China) was used to detect protein concentration. The cell lysates were separated by polyacrylamide gel electrophoresis (4–20% SurePAGE Gel, Genscript, Nanjing, China), and then transferred onto to polyvinylidene difluoride (PVDF) membrane (Bio-Rad, CA, USA). After blocking with 5% nonfat milk in Tris-buffered saline-Tween (TBST) buffer at room temperature for 90 min, the membranes were incubated with the primary antibody at 4 °C overnight. After wash with TBST buffer (10 min × 3), the membranes were incubated with the secondary antibody at room temperature for 1 h. The membranes were examined using the enhanced chemiluminescence kit (Beyotime Biotechnology, Nantong, China). Anti-E-cadherin, anti-Vimentin, anti-N-cadherin, anti-Slug and anti-Snail antibodies were purchased from Cell Signaling Technologies (CST#9782; Boston, MA, USA). Anti-β-actin (ab227387), anti-CD44v6 (ab78960) and anti-FOSL2 (ab124830) antibodies were purchased from Abcam (Cambridge, UK).

### Statistical analysis

SPSS 19.0 software and Prism version 7.0 were used for statistical analyses. Data from three independent experiments were shown as mean ± standard deviation (SD), followed by Student’s t-test, one-way analysis of variance (ANOVA) and chi-square tests. The correlations were analyzed by Pearson correlation. The result with *p-value< 0.05, **p-value < 0.01, or ***p-value < 0.001 was considered as statistically significant.

## Results

### Confirmation of circ0003998 characteristics

**Fig. 1:**
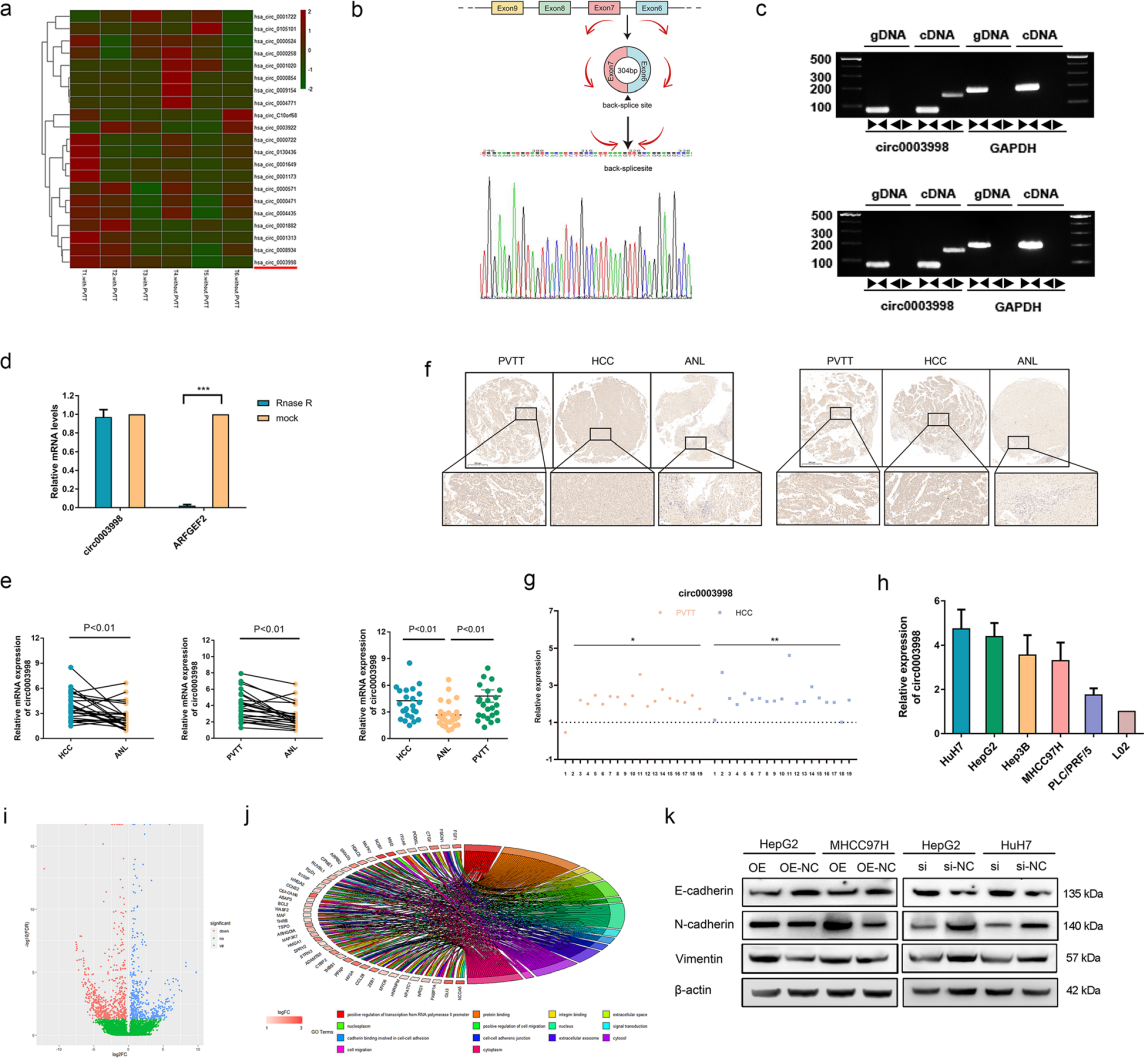
The characteristics and expression of the circ0003998. **a** Cluster heat maps displayed the increase and decrease of circRNAs in 3 HCC patients with PVTT metastasis and 3 HCC patients without PVTT metastasis. Rows indicated circRNAs while columns indicated samples. **b** The scheme illustrated the production of circ0003998 as well as the results of Sanger sequencing. **c** The existence of cric0003998 was validated in HCC cells by agarose gel electrophoresis. GAPDH was used as a negative control. **d** QRT-PCR detected the abundance of circ0003998 and mRNA of ARFGEF2 in HCC cells treated with RNase R, respectively. **e** QRT-PCR showed the relative expression of circ0003998 in HCC, PVTT, and ANL tissues from cohort 1. **f** ISH assay detected the expression of circ0003998 in HCC, PVTT, and ANL tissues from cohort 1; and there were 6 missing value. **g** Semi-quantitative Histochemistry score (H-SCORE) was used to calculate the relative expression of circ0003998 in HCC, PVTT and ANL tissues of TMA-ISH assay. H- SCORE = ∑(percentage of cells of weak intensity× 1) + (percentage of cells of moderate intensity × 2) + (percentage of cells of strong intensity× 3); Relative H-SCORE in PVTT tissues or HCC tissues = (H-SCORE in PVTT tissues or HCC tissues)/(H-SCORE in ANL tissues). **h** QRT-PCR showed the relative expression of circ0003998 in HCC cell lines. **i** The volcano plot showed the expression profile of mRNAs between HCC-OE cells and HCC-NC cells. **j** The Gene Ontology (GO) functional pathways that mRNAs enriched on were showed by Chord Layout. **k** Western blot showed the protein level of EMT markers after over expression or silencing of circ0003998. Data were presented as means ± SD. Student’s t-test was used. *p-value< 0.05, **p-value< 0.01, ***p-value< 0.001. ►◄convergent primer; ◄►divergent primer

We run the RNA-sequencing of HCC samples from patients with PVTT metastasis (PVTT group, *n* = 3) and without PVTT metastasis (HCC group, n = 3). Based on fold change value > 2.0, we identified 13 upregulated, and 8 down regulated circRNAs in the PVTT group as depicted by the heat map (Fig. 1a). Circ0003998 derived from exons (6 to 7) of ARFGEF2 gene were significantly upregulated in the PVTT group (*p* = 0.000000273) than in the HCC group; and therefore, circ0003998 was selected as the candidate circRNA.

To further identify the existence of circ00039998, we amplified circ0003998 by the designed divergent primers and inserted the corresponding PCR products into the T vector for Sanger sequencing. As observed, the corresponding result was consistent with the back-spliced region of circ0003998 provided in circBASE (Fig. 1b). In addition, we designed the convergent primers for the amplification of linear mRNA from exon 6 of ARFGEF2, in order to rule out other potential factors that could induce such head-to-tail splicing [10, 12]. And the results showed, in HCC cells, as opposed to that the linear transcripts could be produced from both cDNA and genomic DNA (gDNA) by convergent primers, circ0003998 was only detected from cDNA instead of gDNA by divergent primers (Fig. 1c). Subsequently, we confirmed the circular characteristics of circ0003998, as the linear transcripts of ARFGEF2 in HepG2 cells could be degraded by the RNase R treatment while circ0003998 could resist to the RNase R treatment (Fig. 1d).

We next detected the expression level of circ0003998 in 25 HCC patients with PVTT metastasis by both qRT-PCR and TMAs-ISH. The results indicated that circ0003998 was significantly upregulated in HCC and PVTT tissues as compared to ANL tissues of HCC patients (cohort 1) (Fig. 1e-g). We further identified that such high expression levels of circ0003998 was correlated with advanced TNM stage (*P* = 0.029) and high serum levels of AFP (*P* = 0.016) in 75 HCC patients (25 samples from cohort 1 and 50 samples from cohort 2) (Table 1). Moreover, the expression level of circ0003998 was also higher in HCC cells than in L02 cells (Fig. 1h).

**Table 1:** Correlation between clinicopathological characteristics and circ0003998 expression levels

Clinicopathological characteristics		circ0003998-low	circ0003998-high	*p*-value
Age(years)				0.797
	≤50	13	11	
	> 50	26	25	
Gender				0.823
	Male	28	25	
	Female	11	11	
MVI				0.630
	Positive	28	24	
	Negative	11	12	
HBsAg				0.701
	Positive	30	29	
	Negative	9	7	
AFP(Ig/L)				0.016^*^
	≤400	29	17	
	> 400	10	19	
Tumor size				0.327
	≤5	15	10	
	> 5	24	26	
TNM stage				0.029^*^
	I + II	25	14	
	III + IV	14	22	

Chi square test was used. MVI, microvascular invasion; AFP, α-fetoprotein; HCC, hepatocellular carcinoma; HBsAg, hepatitis B surface antigen. *p-value< 0.05

### Circ0003998 promotes the EMT of HCC in vitro and in vivo

To explore the biological function of circ0003998 in HCC, we detected the expression profiles of mRNA in 3 paired HCC-OE cells and HCC-NC cells by transcription sequencing (mRNA-seq). Compared with HCC-NC cells, 1027 mRNAs were differentially expressed in HCC-OE cells, among which, expression of 431 mRNAs was upregulated and expression of 596 mRNAs was down regulated, as shown by the volcano plot (Fig. 1i). Gene Ontology (GO) analysis indicated that these mRNAs are enriched in EMT pathways, such as migration, invasion, and focal adhesion (Fig. 1j). Hence, we focused our attention on the role of circ0003998 in EMT of HCC.

**Fig. 2:**
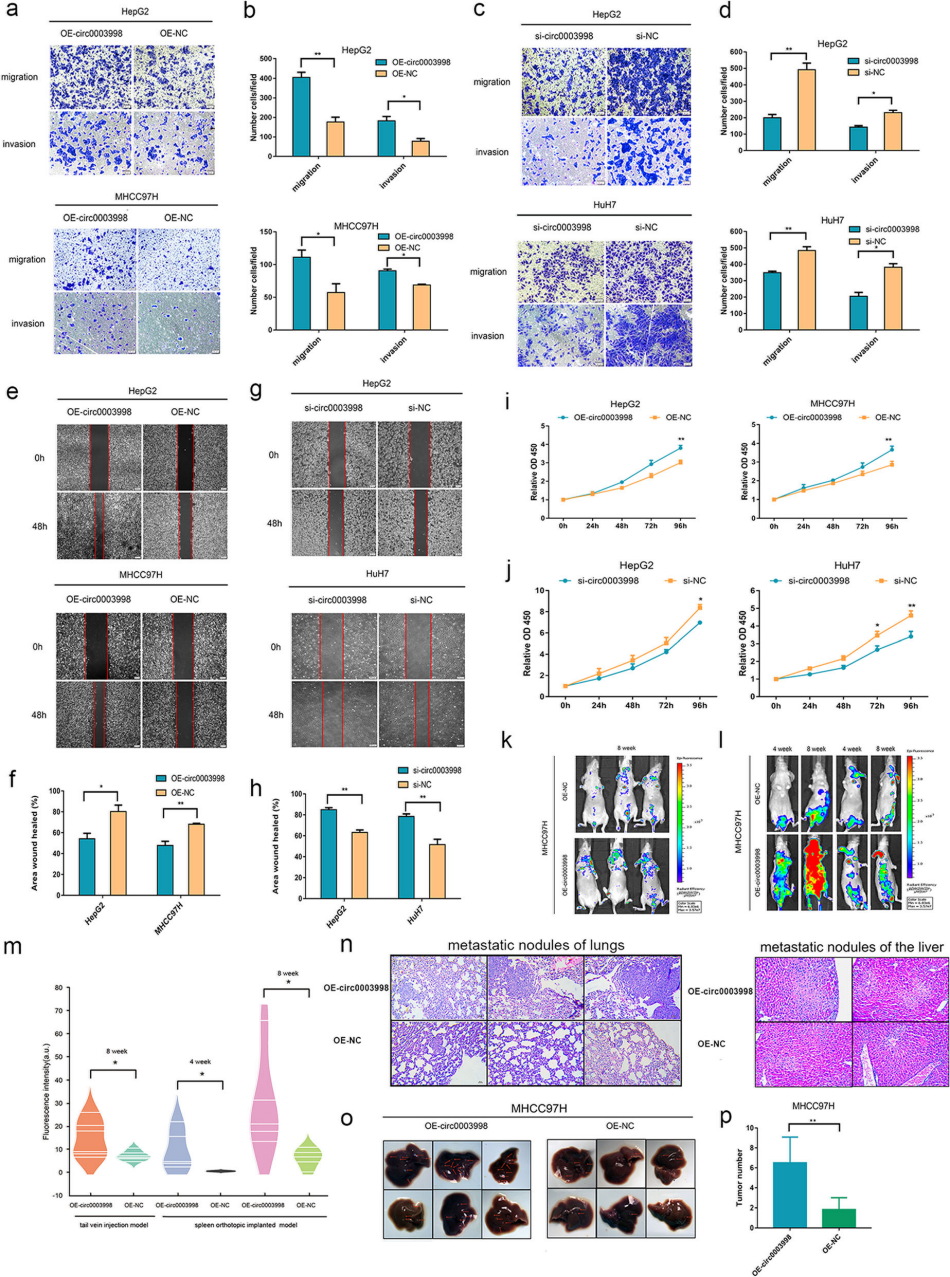
Circ0003998 promoted the EMT of HCC in vitro and in vivo. **a**-**h** Transwell assay (**a**-**d**) and wound healing assay (**e**-**h**) were used to detect the migration and invasion capacities of HCC cells after over expression or silencing circ0003998. Scale bar, 200 μm. **i**-**j** Cell Counting Kit-8 assay was performed to assess cell growth. **k** GFP-signal intensities showed the metastatic rate of lung metastatic mice model after the tail vein injection for 8 weeks. **l** GFP-signal intensities showed the metastatic rate in the liver metastatic model after spleen injection for 4 weeks and 8 weeks. **m** The GFP-signal intensities were calculated in the lung metastatic mice model and liver metastatic mice model. **n** Representative images of H&E staining showed the metastatic lesions in both the lung metastatic mice model and liver metastatic model after the HCC cells injection for 8 weeks. **o** Representative images of metastatic foci were indicated by red arrows in the liver metastatic model after the HCC cells injection in spleen for 8 weeks. **p** The number of metastatic foci in the liver metastatic model was counted after spleen injection for 8 weeks. All data are presented as means ± SD of three independent experiments; Student’s t-test was used. *p < 0.05, **p < 0.01, ***p < 0.001

We infected MHCC97H and HepG2 cells (MHCC97H-OE/NC and HepG2-OE/NC) with OE/NC -circ-vector and transfected si/NC-circ0003998 into HuH7 and HepG2 cells (HuH7-si/NC and HepG2-si/NC) (Fig. S1a). Firstly, we found that over expression of circ0003998 inhibits the protein level of E-cadherin and enhances the protein level of Vimentin, N-cadherin, Snail and Slug; while down expression of circ0003998 showed the opposite results (Fig. 1k, Fig. S1b). Secondly, we explored the role of circ0003998 in EMT of HCC by in vitro cell phenotypic assays. Transwell assay indicated that over expression of circ0003998 and knockdown of circ0003998 expression respectively increases and decreases cell metastasis (Fig. 2a-d). Wound healing assay showed that the mobility of HCC cells is significantly enhanced when circ003998 was over expressed, but markedly impaired when circ0003998 expression was knocked down (Fig. 2e-h). Furthermore, the results of CCK8 assay demonstrated that ectopic expression of circ0003998 promotes the proliferation of HCC cells, whereas the down regulation of circ0003998 restrained the proliferation of HCC cells (Fig. 2i-j). Thirdly, we applied nude mice model with lung metastatic or with liver metastatic to study the role of circ0003998 in EMT of HCC in vivo. By monitoring the fluorescence value of GFP, we found that circ0003998 significantly accelerated the metastasis of HCC into both lung and liver. (Fig. 2k-m). Furthermore, haematoxylin and eosin (H&E) staining suggested that over expression of circ0003998 remarkably promoted metastasis of HCC into both lung and liver (Fig. 2n). And the number of metastatic foci was also increased with the over expression of circ0003998 in liver metastatic mice model (Fig. 2o-p). These results indicated that circ0003998 could play an oncogenic role in the EMT in HCC both in vitro and in vivo.

### Circ0003998 function as a sponge for miR-143-3p

**Fig. 3:**
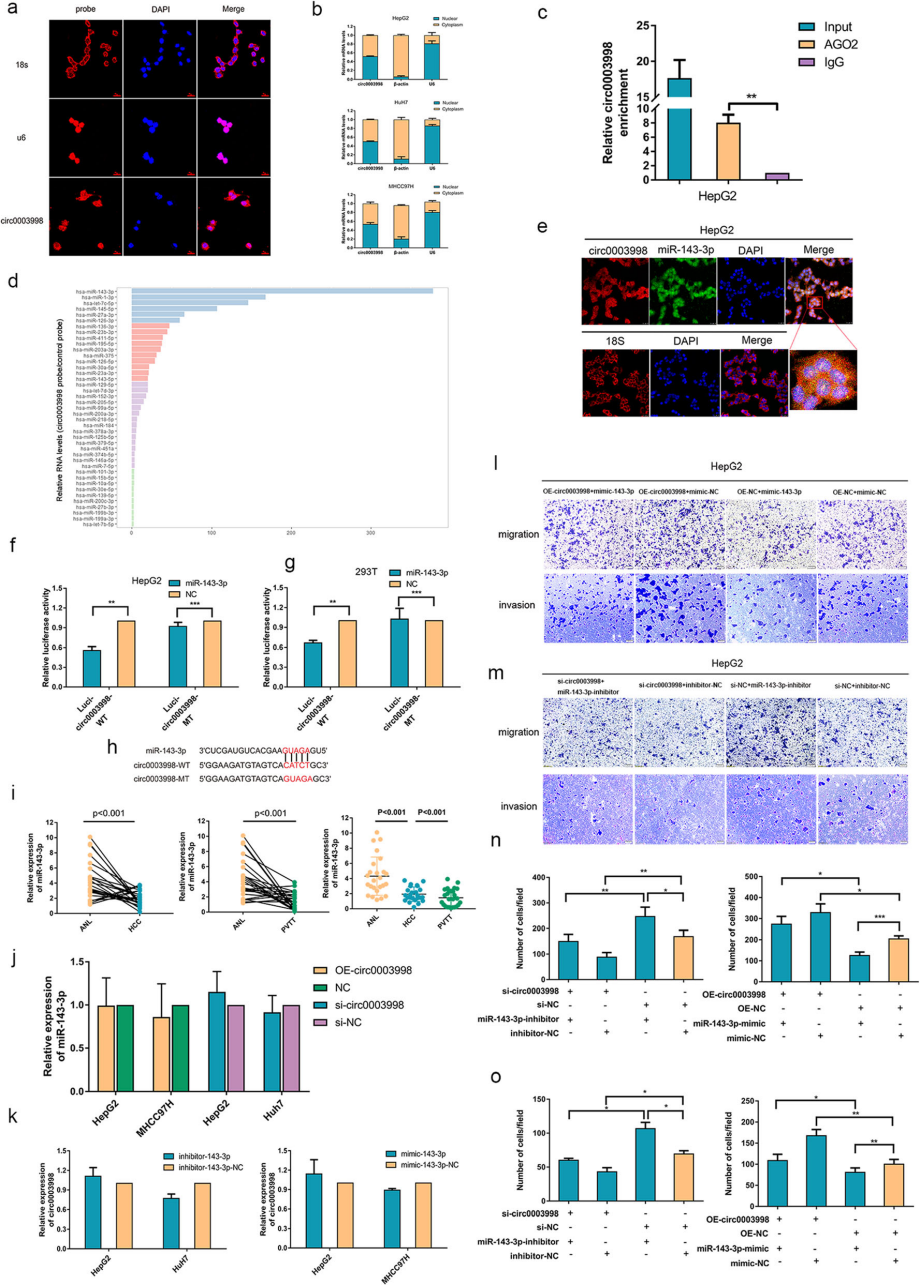
Circ0003998 functioned as a sponge for miR-143-3p. **a** FISH assay was used to detect the intracellular location of circ0003998; 18S and U6 were applied as positive controls in the cytoplasm and nucleus, respectively. Nuclei were stained with DAPI. **b** The intracellular location of circ0003998 was detected by separating the nuclear and cytoplasmic fractions of HCC cells and was assessed with nuclear control U6 and cytoplasmic control β-actin by qRT-PCR. **c** Anti-AGO2 RIP was performed in HepG2 cells. **d** RNA-pull down was performed in HepG2 cells using a circ0003998-specific probe and control probe, respectively; and the enrichment of miRNAs was detected by miRNA-seq. **e** FISH was performed to observe the co-localization between circ0003998 (red) and miR-143-3p (green) in HepG2 cells (magnification, × 400, scale bar, 25 μm). **f**-**g** The luciferase activities were detected in HepG2 cells and HEK293T cells after transfection with circ003998-WT or circ0003998-Mut and miR-143-3p mimics or miR-NC, respectively. **h** A schematic drawing showed the putative binding sites between miR-143-3p and circ0003998. **i** Relative expression of miR-143-3p in HCC tissues, PVTT tissues, and ANL tissues in cohort 1 (*n* = 25) were determined by qRT-PCR. **j** The relative expression of miR-143-3p was detected by qRT-PCR after over expression or silencing circ0003998 in HepG2 cells. **k** The relative expression of circ0003998 was detected by qRT-PCR after the transfection of miR-143-3p mimic or inhibitor. **l**-**o** Rescue transwell assays of migration and invasion were performed after transfection with indicated vectors, miR-143-3p mimic or inhibitors (magnification, × 100, scale bar, 100 μm). Data are presented as means ± SD; Student’s t-test was used. *p-value< 0.05, **p-value< 0.01, ***p-value< 0.001

Given that circRNAs could function as sponges for miRNAs and the latter was mostly located in the cytoplasm, we firstly tried to explore the subcellular localization of circ0003998 in the HCC cells. By FISH assay, we found circ0003998 in both cytoplasm and nucleus (Fig. 3a), which was further verified by quantitatively detecting the expression of circ0003998 in the nuclear and cytoplasmic fractions in HCC cell lines (Fig. 3b). Considering that miRNAs silence the expression of targeted genes by binding with argonaute 2 (AGO2) [13], secondly, we run RIP assay in HepG2 cells using AGO2 antibodies. We found that circ0003998 was significantly enriched in the presence of AGO2 antibodies, suggesting that circ0003998 could act as the platform for the binding between miRNAs and AGO2 (Fig. 3c). Taken together, our data demonstrated the potentiality of circ0003998 to sponge miRNAs.

Following, we run the pull-down assay using specific probes against circ0003998 to screen the specific miRNA that possibly sponged by circ0003998. The precipitation complexes obtained from HCC cells were analyzed by miRNAs sequencing, and the result indicated that miR-143-3p is significantly enriched and it might be the potential targets of circ0003998 (Fig. 3d). We further confirmed this result by FISH assay that most circ0003998 (red) and miR-143-3p (green) are co-located in the cytoplasm in HCC cells (Fig. 3e, Fig. S1c). Additionally, dual-luciferase reporter assay in both HEK293T cells and HepG2 cells also indicated the consistent result that miR-143-3p mimic could inhibit the luciferase activity of wide-type circ0003998 vector (circ0003998-WT vector) as opposed to mutant-type circ0003998 vector (circ0003998-Mut vector) (Fig. 3f-h).

Next, we detected the correlation between the expression of circ0003998 and miR-143 -3p. The expression levels of miR-143-3p were down regulated in HCC and PVTT tissues as compared to ANL tissues (cohort 1), respectively (Fig. 3i). Moreover, the expression level of miR-143-3p showed no significant changes after over expression or silencing of circ0003998 in HepG2 cells (Fig. 3j), while the expression level of circ0003998 showed no significant changes after transfection with miR-143-3p mimic or inhibitor (Fig. 3k). Additionally, the functional rescue experiments indicated that miR-143-3p mimics could abolish the invasion-promoting effects of circ0003998 over expression, whereas miR-143-3p inhibitors could reverse the invasion- suppressing effects of silencing circ0003998 (Fig. 3l-o). These findings suggested that circ0003998 directly sponges with miR-143-3p as the ceRNA in HCC, and circ0003998 is neither induced into the degradation of miR-143-3p nor regulated by miR-143-3p.

### Circ0003998 promotes the EMT in HCC cells through miR-143-3p/FOSL2 pathway

**Fig. 4:**
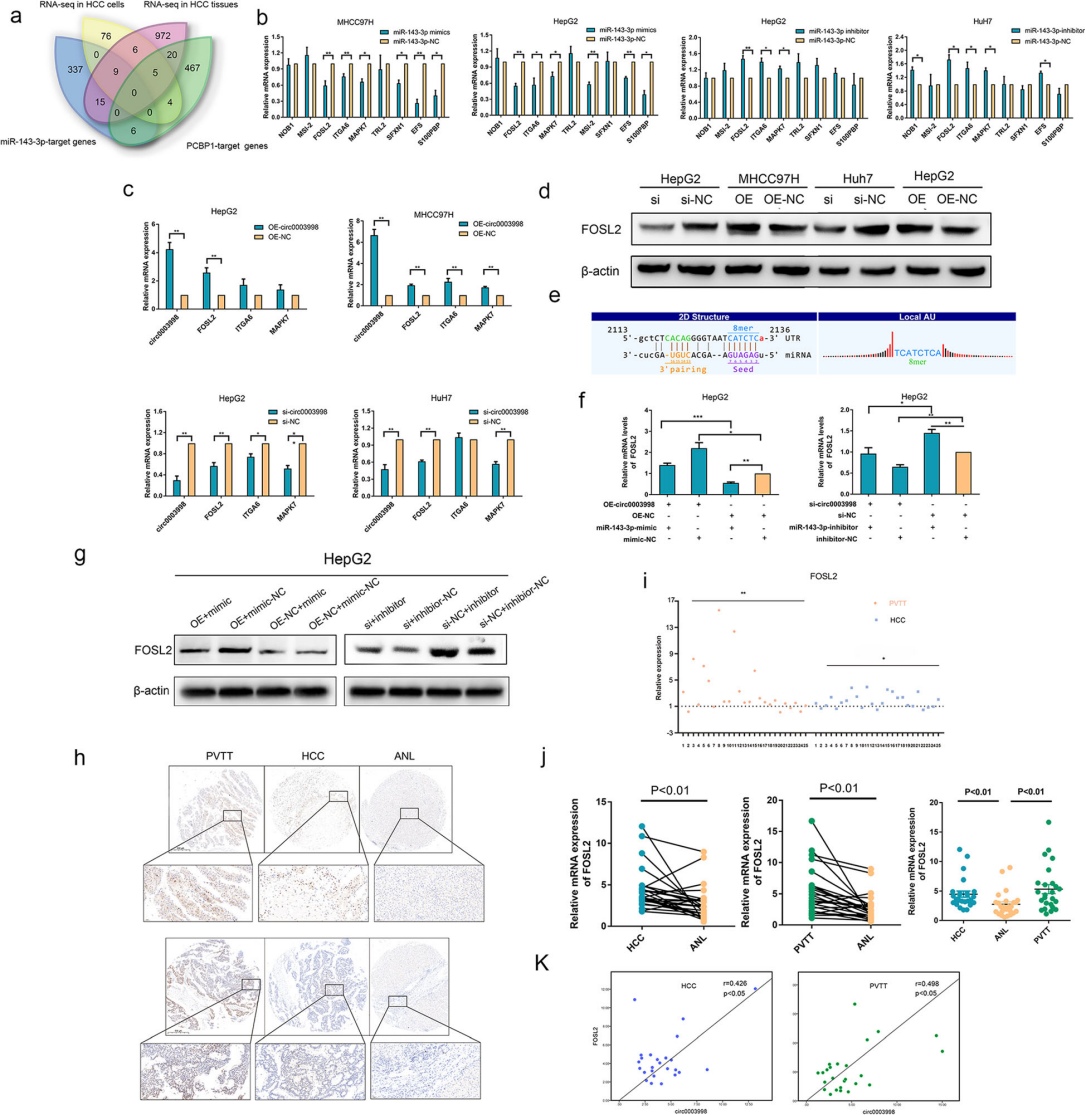
Circ0003998 promoted the EMT in HCC cells through the miR-143-3p-FOSL2 pathway. **a** Venn diagram showed the differentially expressed genes in HCC cells (HCC-OE cells/HCC-NC cells) and HCC tissues from patients with/without PVTT metastasis by RNA-sequencing, as well as the predicted target genes of miR-143-3p or PCBP1. **b** The qRT-PCR showed the mRNA level change of the predicated targets with miR-143-3p mimic or miR143-3p inhibitor in HCC cells. **c** The qRT-PCR showed the mRNA level change of the predicated targets after over expression or silencing of circ0003998. **d** Western blot showed protein levels of FOSL2 after over expression or silencing of circ0003998. **e** The miR-143-3p binding sites on circ0003998 were predicted by target Scan and miRanda. **f-g** Relative expression of FOSl2 was detected in circ0003998-OE HepG2 cells transfected with miR-143-3p mimics and circ0003998-silencing HepG2 cells transfected with miR-143-3p inhibitors. **h** ISH assay detected the expression of FOSL2 in HCC, PVTT, and ANL tissues from cohort 1. **i** Semi-quantitative H-SCORE was used to calculate the relative expression of FOSL2 in HCC, PVTT and ANL tissues by TMA-ISH assay. H-SCORE = ∑ (percentage of cells of weak intensity× 1) + (percentage of cells of moderate intensity× 2) + (percentage of cells of strong intensity× 3); Relative H-SCORE in PVTT tissues or HCC tissues = (H-SCORE in PVTT tissues or HCC tissues)/(H-SCORE in ANL tissues). **j** QRT-PCR detected the relative mRNA levels of FOSL2 in HCC, PVTT, and ANL tissues in cohort 1. **k** The correlation between the expression of circ0003998 and FOSL2 was analyzed in HCC tissues and PVTT tissues in cohort 1. Data were presented as means±SD; *n* = 3. Student’s t-test was used. The correlation was measured by Pearson correlation analysis. *p-value< 0.05, **p-value< 0.01, ***p-value< 0.001

Given above results, we further screened the targeted genes of miR-143-3p which play the oncogenetic role in the EMT of HCC and might be released due to the sponge between circ0003998 and miR-143-3p. Firstly, we predicted the possible target genes of miR-143-3p in TargetScan (http://www.targetscan.org/vert_72/) and miRanda (http://miranda.org.uk/) (Additional file 3). Secondly, we analyzed the mRNA expression profile in both HCC cells (circ0003998-OE vs. circ0003998-NC) and HCC tissues (with/without PVTT metastasis) by mRNA-seq. According to the prediction of target genes of miR-143-3p and the results of mRNA-seq, we screened 9 differentially expressed genes (NOB1, MSI-2, FOSL2, ITGA6, MAPK7, TRL2, S100PBP, SFXN1, and EFS) (Fig. 4a).

Among the 9 candidate genes, the mRNA expression of FOSL2, ITGA6, MAPK7 were significantly down regulated by miR-143-3p mimics and upregulated by miR-143-3p inhibitors in HCC cells (Fig. 4b). Then the mRNA expression of these three targets (FOSL2, ITGA6, MAPK7) were detected after over expressing or silencing circ0003998 and we found that the mRNA expression of FOSL2 is significantly enhanced in MHCC97H-OE and HepG2-OE cells whereas decreased in HuH7-si and HepG2-si cells (Fig. 4c). Moreover, the protein expression of FOSL2 altered with the same trend as the mRNA levels under the regulation of circ0003998 (Fig. 4d). Meaningfully, FOSL2 has been identified as the promoter of EMT on TGF-β/Smad3 signaling pathway in advanced cancers [14–16]. Thus we presumed that circ0003998 promoted the EMT of HCC by protecting FOSL2 from down regulating by miR-143-3p, which was verified by means of the following assays.

Firstly, we predicted the binding sites of miR-143-3p with FOSL2 by TargetScan and miRanda (Fig. 4e). Secondly, we identified that circ0003998 increased the expression of FOSL2 in HepG2 cells, which was reversed by exogenous miR-143-3p mimics; while the silence of circ0003998 suppressed the expression of FOSL2 in HepG2 cells, which was retarded by miR-143-3p inhibitors (Fig. 4f-g). Thirdly, the functional rescue experiments consistently showed that FOSL2 is the downstream target gene of circ0003998 /miR-143- 3p axis to promote migration of HCC (Fig. S1d). Finally, we detected the the correlation of expression between circ0003998 and FOSL2 in HCC tissues (cohort 1). The expression levels of FOSL2 were highly upregulated in HCC tissues and PVTT tissues as compared to ANL tissues by both ISH-TMAs and qRT-PCR analysis (Fig. 4h-j). And by Pearson correlation analysis, the expression levels between FOSL2 and circ0003998 showed a positive association in both HCC tissues and PVTT tissues, respectively (Fig. 4k). Thus, our study suggested that circ0003998 functioned as a ceRNA by adsorption of miR-143-3p to release FOSL2, which promoted EMT of HCC.

### Circ0003998 binds to poly(rC) binding protein 1 (PCBP1)

**Fig. 5:**
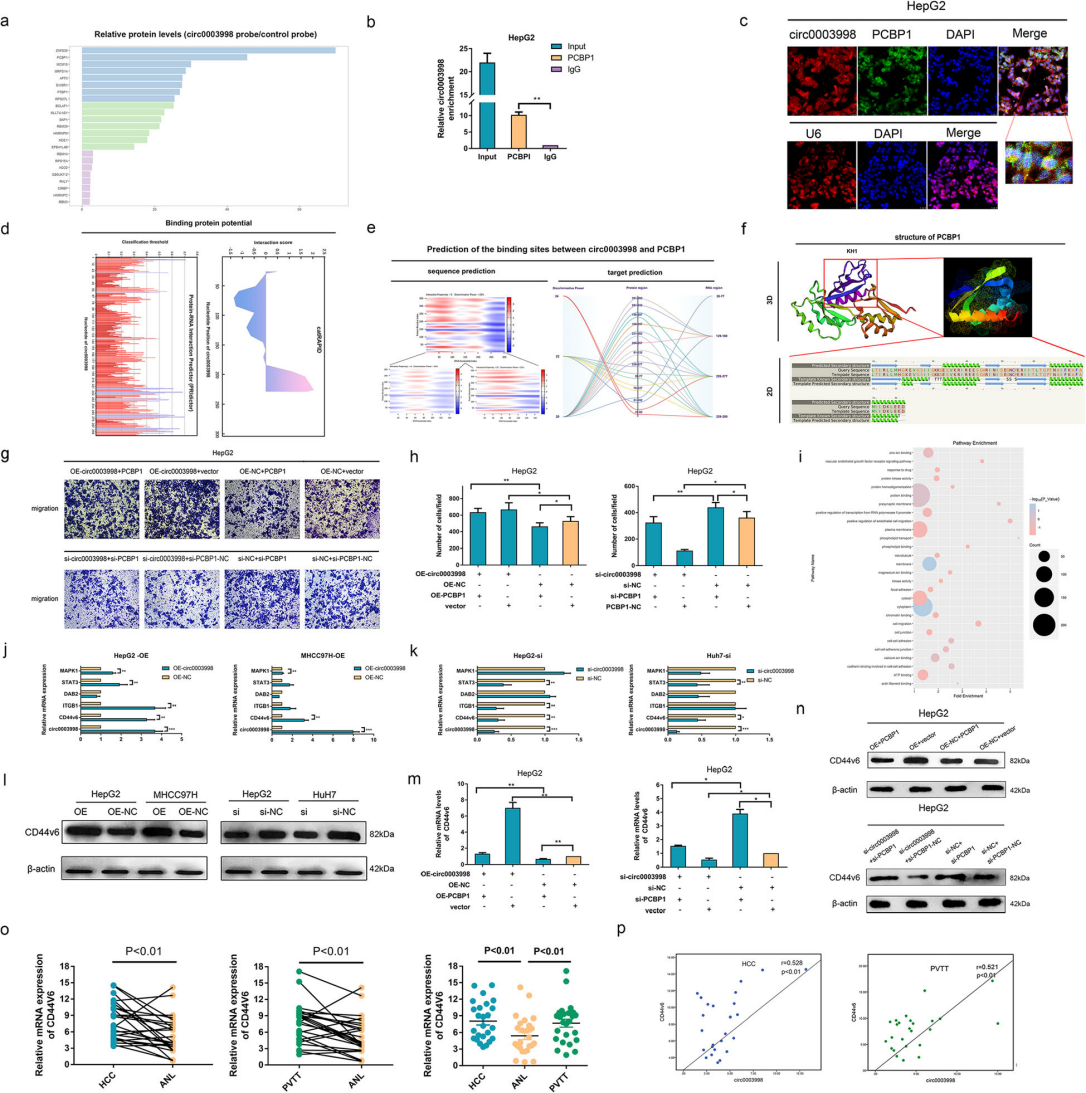
Circ0003998 promoted the EMT in HCC cells through PCBP1/CD44v6 pathway. **a** Protein pull-down experiment was performed using the circ0003998-specific probe and control probe; and the enrichment of protein was detected by the protein spectrum. **b** RIP experiment was performed with anti-PCBP1 antibody and negative control IgG, followed by qRT-PCR to detect the expression of circ0003998. **c** FISH was performed to observe the cellular location of circ0003998 (red) and PCBP1 (green) in HepG2 cells (magnification, × 400, scale bar, 25 μm). **d**-**e** The binding sites between circ0003998 and PCBP1 were predicted online. **f** The structure of the KH1 of PCBP1 was modeled. **g**-**h** Rescue transwell assay was performed after transfection with indicated vectors, OE-PCBP1 or si-PCBP1 (magnification, × 100, scale bar, 100 μm). **i** GO analysis showed the enriched pathway of PCBP1 target genes. **j**-**k** QRT-PCR showed the mRNA level of the targets of PCBP1 after over expression or silencing of circ0003998. **l** Western blot showed the protein level of the CD44v6 after over expression or silencing of circ0003998. **m**-**n** Relative expression of CD44v6 was detected in circ0003998-OE HepG2 cells transfected with OE-PCBP1 and circ0003998-silencing HepG2 cells transfected with si-PCBP1. **o** QRT-PCR detected the relative mRNA levels of CD44v6 in HCC, PVTT, and ANL tissues in cohort 1. **p** The correlation between the expression of circ0003998 and CD44v6 was analyzed in HCC tissues and PVTT tissues in cohort 1. The correlation was measured by Pearson correlation analysis. Data are presented as means ± SD; n = 3. Student’s t-test was used. *p < 0.05, **p < 0.01, ***p < 0.001

Results from above study revealed that circ0003998 played stimulating role in EMT of HCC by sponging with miR-143-3p. Meanwhile, considering that the interaction with RNA binding proteins (RBPs) is an important part of circRNA function [17], it remained to reveal whether circ0003998 could bind with RBPs to participate in the EMT of HCC. Firstly we performed pull-down assay and protein spectrum to identified the specific RBP bound by circ0003998 (Fig. 5a). And poly(rC) binding protein 1 (PCBP1) was selected as the binding protein of circ0003998 as it was not only precipitated by circ0003998-specific probes but also received the highest prediction score in catRAPID (Additional file 4). Secondly, RIP experiments in HepG2 cells verified the results of pull down assay as it showed that circ0003998 is considerably enriched by specific antibodies against PCBP1 compared with IgG (Fig. 5b). Thirdly, FISH assay in HepG2 cells also confirmed that circ0003998 (red) and PCBP1 (green) were co-located in the nucleus and cytoplasm, especially in the nuclear membrane (Fig. 5c, Fig. S1c).

**Fig. 6:**
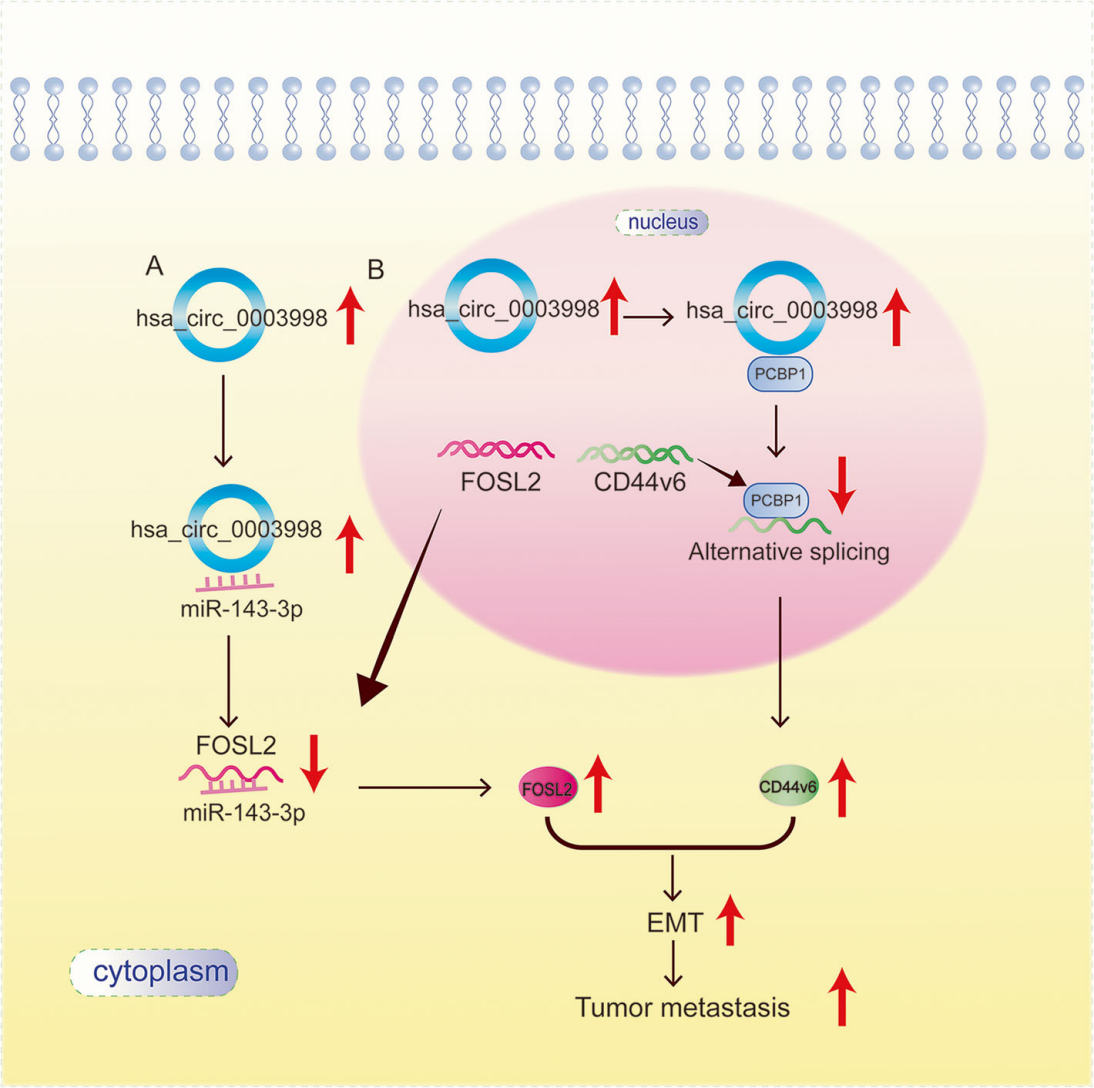
Potential schematic pathway illustrated the role of circ0003998 in the process of EMT in HCC

Further, we predicted the potential binding sites between circ0003998 and PCBP1. Firstly, we analyzed the nucleotides sequence of circ0003998 by online catRAPID and PRIdictor (http://www.rna-society.org/rnainter/php/PRIdictor.php), which indicated that nucleotides at 220–300 with a high potential to bind with protein (Fig. 5d). Secondly, we predicted that nucleotides at 220–280 of circ0003998 and polypeptide at 31–82 of PCBP1 could be the potential binding sites of each other by online catRAPID (Fig. 5e). Thirdly, we analyzed that the polypeptide at 31–82 of PCBP1 were belonged to its K homology 1 (KH1) and we modeled the 3D structure of KH1 (Fig. 5f). Moreover, the results in functional rescue assays also consistently confirmed circ0003998 could bind with PCBP1, for PCBP1 could reverse the role of circ0003998 on the migration ability in HCC cells. (Fig. 5g-h).

### Circ0003998 promotes the EMT in HCC cell through PCBP1/CD44v6 pathway

Since PCBP1 is recognized as a tumor suppressor in previous studies [18–20], we hypothesized that function of PCBP1 could be inhibited when bind to circ0003998, which thereby promote EMT of HCC. Firstly, Gene Transcription Regulation Database (GTRD, http://gtrd.biouml.org/) were used to predict the possible target genes of PCBP1 (Additional file 5), which were significantly enriched in the EMT-related pathway by GO analysis (Fig. 5i). Secondly, we measured the expression levels of five target genes of PCBP1 (CD44v6, STAT3, MAPK1, ITGB1, DAB2) based on the results of according to the analysis of mRNA sequencing in HCC and tissues and results of previous studies (Additional file 6, Fig. 4a). Among the five potential target genes, the mRNA expression levels of CD44v6 were significantly upregulated in HepG2-OE and MHCC97H-OE cells, while down regulated in HuH7-si and HepG2-si cells (Fig. 5j-k). In addition, previous study reported that PCBP1 regulated the expression of DAB2 at translation level [21]. However, the protein level of DAB2 showed no significant changes with circ003998 upregulation or silencing (Fig. S1e).

Given above results and the promoting-role of CD44v6 in EMT in cancers [22, 23], we further studied that whether circ0003998 promoted the EMT of HCC by protecting the CD44v6 from down regulation by PCBP1. Firstly, we found that the protein levels of CD44v6 was significantly upregulated in HepG2-OE and MHCC97H-OE cells, while it was down regulated in HuH7-si and HepG2-si cells (Fig. 5l). Next, rescue assay showed that PCBP1 could abolish circ000398 regulation on both the mRNA and proteins expression level of CD44v6 in HCC cells (Fig. 5m-n). Then the mRNA levels of CD44v6 were highly upregulated in 25 pairs of HCC and PVTT tissues as compared to ANL tissues (Fig. 5o). Pearson correlation analysis indicated that the expression level of CD44v6 were positively associated with that of circ0003998 both in HCC and PVTT tissues (Fig. 5p). These findings further demonstrated that circ0003998 inhibited HCC metastasis through circ0003998/PCBP1/CD44v6 axis.

## Discussion

More and more circRNAs are discovered using high-throughput sequencing techniques and bioinformatics analysis. Previous study has showed that circ0003998 could promote cell proliferation and invasion by targeting miR-326 in non-small cell lung cancer [24]. In this study, our data for the first time identified that circ0003998 could promote EMT in HCC by in vitro functional assays and in vivo mice models; and meanwhile circ0003998 was upregulated in HCC tissues and PVTT tissues, which were correlated with advanced TNM stage and high serum levels of AFP. Furthermore, our previous study has confirmed that expression of circ0003998 is also upregulated in plasma in HCC, which is significantly correlated with poor overall survival (OS) in HCC patients; and therefore, circ0003998 could be used as independently prognostic factors for poor OS in HCC [25].

Mechanically, we identified that circ0003998 could sponge miR-143-3p, the suppressor of proliferation, invasion, and metastasis in multiple cancers [26–28] as well as the inhibitor of the EMT-related genes [29–31]. Furthermore, there was no significant correlation between the expression of miR-143-3p and circ0003998. Previous reports showed that ceRNAs could regulate the activity of miRNAs; however, whether ceRNAs regulate the expression levels of miRNAs might rely on the cellular context [32, 33]. Our data further showed that circ0003998 could sponge with miR-143-3p to increase the expression of FOSL2 and thereby promoted the EMT in HCC and it has been consistently showed that FOSL2 is the target gene of miR-143-3p in osteosarcoma in previous study [34].

The tertiary structures of circRNAs result in higher protein adsorbing capacity than those of linear RNA sequences. As such, the circRNA-interacting RBPs serves as an essential molecular action mode in genesis, translation, transcriptional regulation of target genes, and extracellular transport [35]. PCBP1 as one of the the RBPs inhibited the tumor formation and metastasis by translation silencing, mRNA alternative splicing, or transcription of carcinogenic genes [36]. Meaningfully, PCBP1 is reported to participate in EMT pathways in cancer, especially in the TGF-β pathway [37–39]. And our data showed that circ0003998 bound to PCBP1 to increase the expression of CD44v6 and thereby promoted the EMT in HCC, which was consistent with former studies that PCBP1 regulated alternative splicing of the CD44v6 and thereby inhibited the invasion in HCC [40].

Previous study showed that PCBP1 regulated DAB2 in the translation process [21]. However, there were no significant changes in the protein level of DAB2 with circ0003998 regulating in our data. The sponging affinity of circRNAs with RBPs was not the same as that between circRNAs and miRNAs, and circRNAs absorbing RBPs might be more complicated than expected [41]. Thus further research is necessary to understand whether circ0003998 inhibits the translation regulating-role of PCBP1 or whether circ0003998 induces the degradation of PCBP1.

## Conclusions

We identified that circ0003998 expression is remarkably upregulated in HCC, PVTT tissues, and which is correlated with advanced TNM stage and high serum levels of AFP. Circ0003998 locates in both cytoplasm and nucleus and it regulates EMT of HCC by both circ0003998/miR-143 -3p/FOSL2 axis and circ0003998/PCBP1/CD44v6 axis (Fig. 6).

## Supplementary information

**Additional file 1.** The identification of HCC cells in this study by short tandem repeat.

**Additional file 2.** The primer details and siRNA sequence used in this study.

**Additional file 3.** Predicted target genes for hsa-miR-143-3p in TargetScan and miRanda.

**Additional file 4.** Prediction score of RBPs binding to circ0003998 using the online catRAPID algorithm.

**Additional file 5.** Predicted target genes of PCBP1 in Gene Transcription Regulation Database.

**Additional file 6.** Tumor promoter in HCC which are targets of PCBP1.

**Additional file 7: Fig. S1** (a) QRT-PCR detected the relative expression level of circ0003998 after over expressing or silencing circ0003998 in HCC cells. (b) The relative protein expression level of Snail and Slug in the HCC cells with circ0003998 over expression and silencing. (c) FISH was performed to observe the cellular location of circ0003998 (red), miR-143-3p (green) and PCBP1 (green) in HuH7 cells (magnification, × 400, scale bar, 25 μm). (d) Rescue transwell assay was performed after trans-fection with indicated vectors, OE-FOSL2 or si-FOSL2 (magnification, × 100, scale bar, 100 μm). (e) The relative protein expression level of DAB2 in HCC cells with circ0003998 over expression and silencing. (f) The relative mRNA expression of circ003998, FOSL2 and CD44v6 in HCC and PVTT tissues. *p-value< 0.05, **p-value< 0.01, ***p-value< 0.001.

## Data Availability

The data used and analyzed during the current study are available from the corresponding author on reasonable request.

## Abbreviations

circRNAs: Circular RNAs
miRNAs: microRNA
HCC: Hepatocellular carcinoma
circ0003998: has_circ_ 0003998
PVTT: Portal vein tumor thrombus
PCBP1: PCBP1-poly(rC) binding protein 1
EMT: Epithelial to mesenchymal transition
RNA-seq: RNA-sequencing
ARFGEF2: ADP ribosylation factor guanine nucleotide exchange factor 2
miR-143-3p: microRNA-143-3p
ANL: Adjacent non-cancerous liver tissues
STR: short tandem repeat
RNA-seq: RNA-sequencing
qRT-PCR: Quantitative polymerase chain reaction
PCR: polymerase chain reaction
FISH: Fluorescence in situ hybridization
TMA: Tissue microarray
H&E: Haematoxylin and eosin
PBS: Phosphate buffer saline
siRNAs: Small interfering RNAs
CCK-8: Cell counting kit-8 kit
RIP: RNA immunoprecipitation
AGO2: Argonaute2
PVDF: Polyvinylidene difluoride
TBST: Tris-buffered saline-Tween
ANOVA: One-way analysis of variance
GO: Gene Ontology
FOSL2: FOS like 2, AP-1 transcription factor subunit
CD44v6: Variant isoform 6 of CD44
NC: Negative control
cDNA: Complementary DNA
gDNA: genomic DNA
OE: Over expression of circ0003998
si: Silencing of circ0003998
DAB2: Disabled2
