# Supplementary figures and images for: Hsa_circ_0003998 promotes epithelial to mesenchymal transition of hepatocellular carcinoma by sponging miR-143-3p and PCBP1
Source: J Exp Clin Cancer Res. 2020 Jun 17;39:114. doi: 10.1186/s13046-020-01576-0 (PMC7302140; doi:10.1186/s13046-020-01576-0)

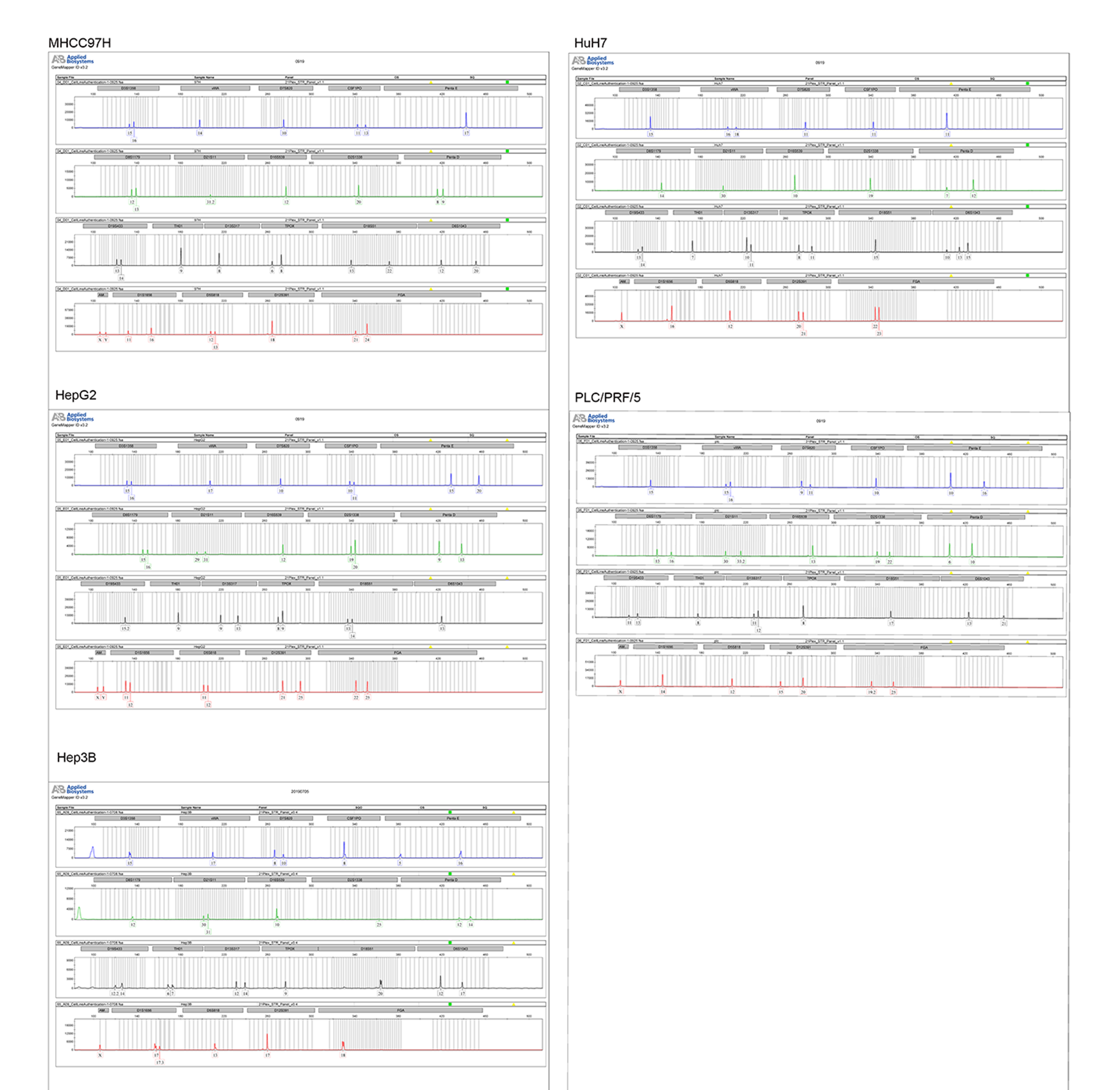

Supplement: Supplementary file 1 — Additional file 1. The identification of HCC cells in this study by short tandem repeat. [file 13046_2020_1576_MOESM1_ESM.tif]

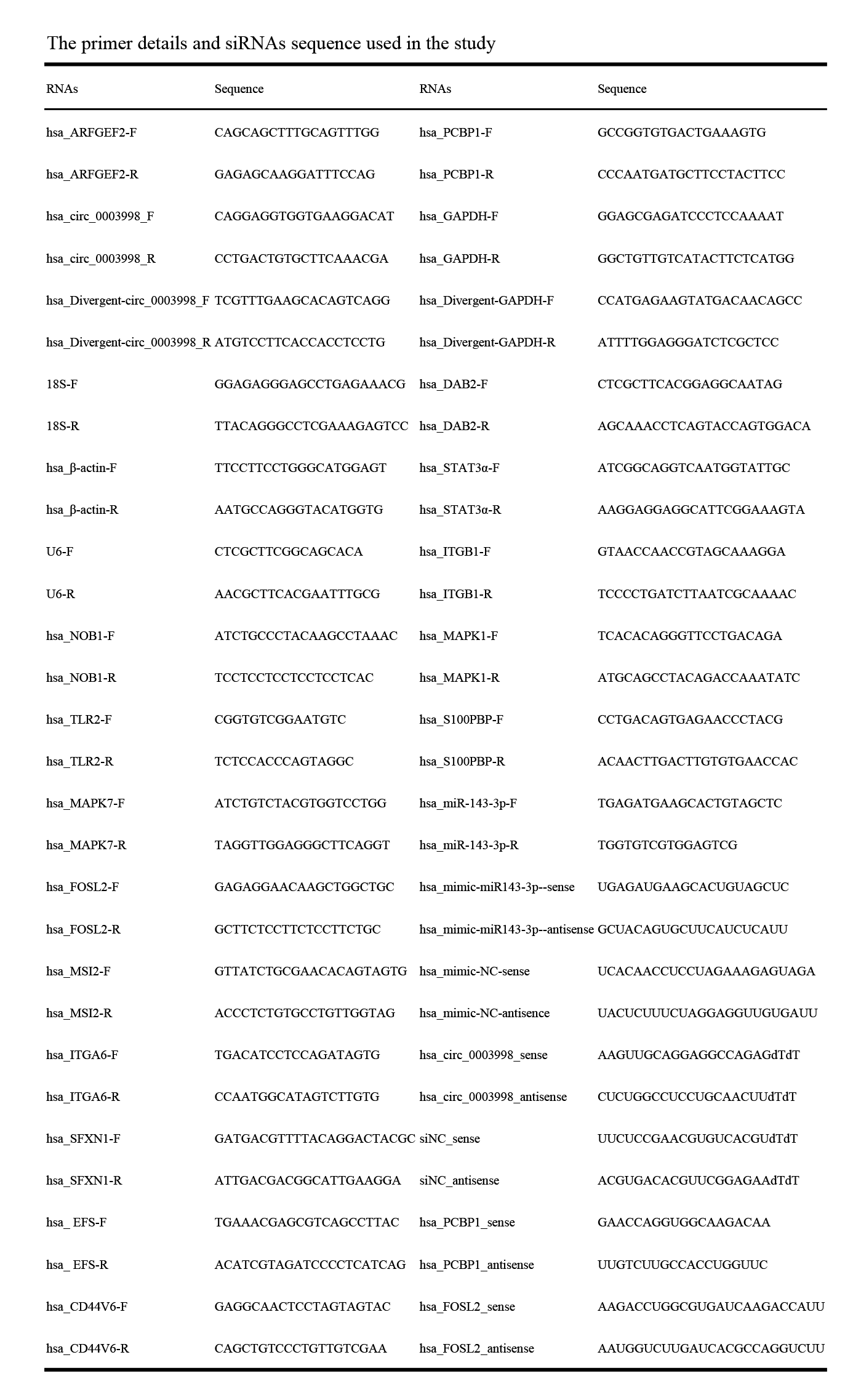

Supplement: Supplementary file 2 — Additional file 2. The primer details and siRNA sequence used in this study. [file 13046_2020_1576_MOESM2_ESM.tif]

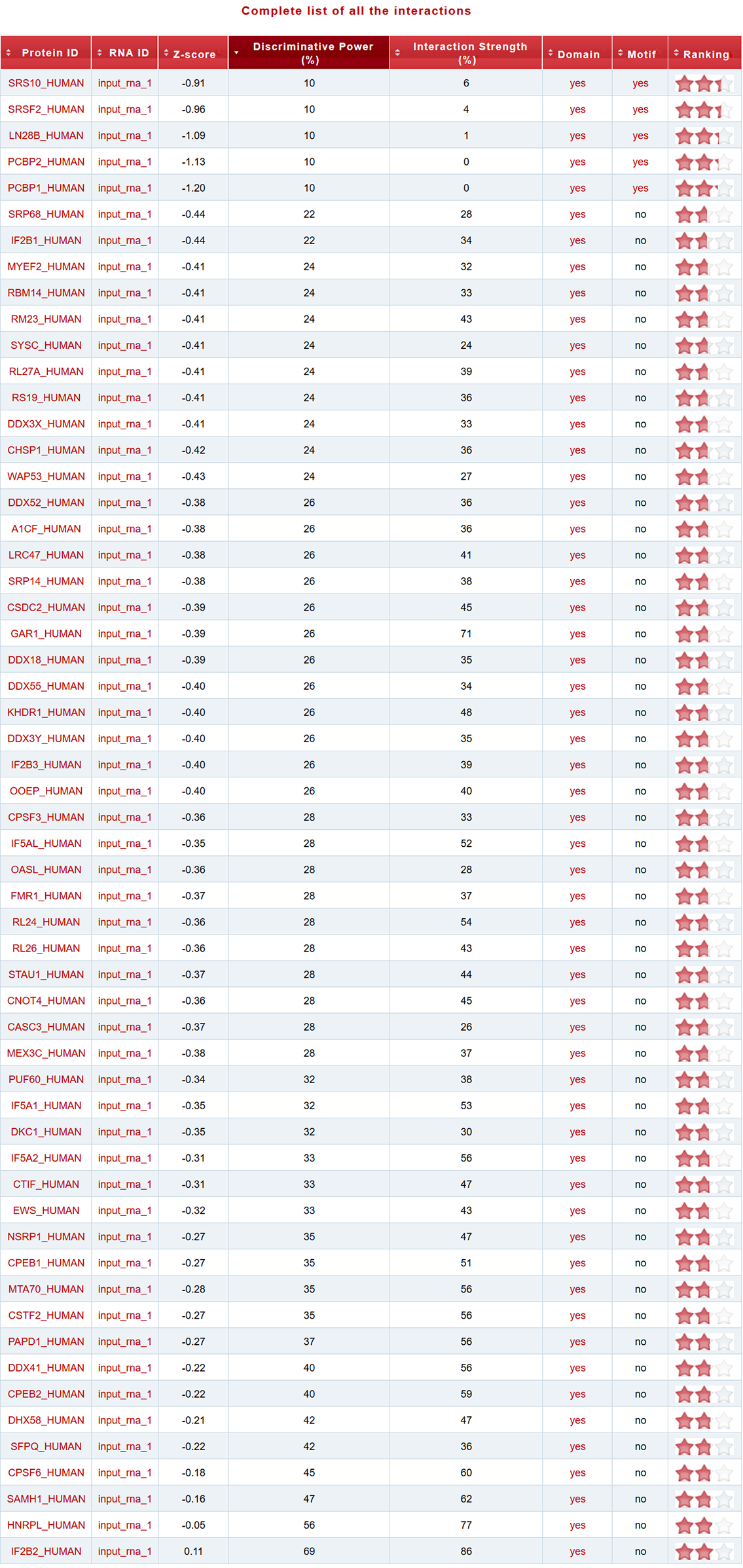

Supplement: Supplementary file 4 — Additional file 4. Prediction score of RBPs binding to circ0003998 using the online catRAPID algorithm. [file 13046_2020_1576_MOESM4_ESM.tif]

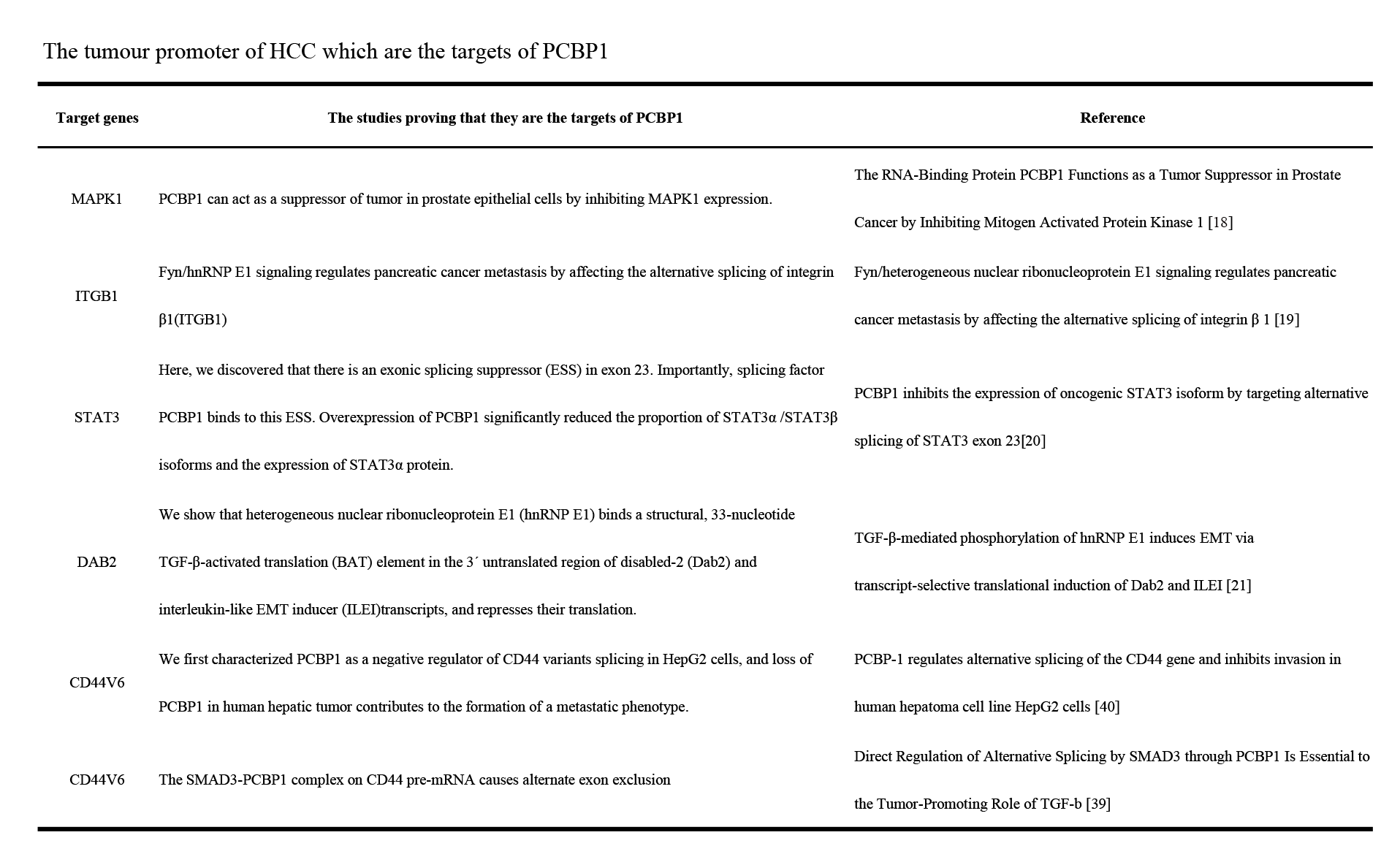

Supplement: Supplementary file 6 — Additional file 6. Tumor promoter in HCC which are targets of PCBP1. [file 13046_2020_1576_MOESM6_ESM.tif]

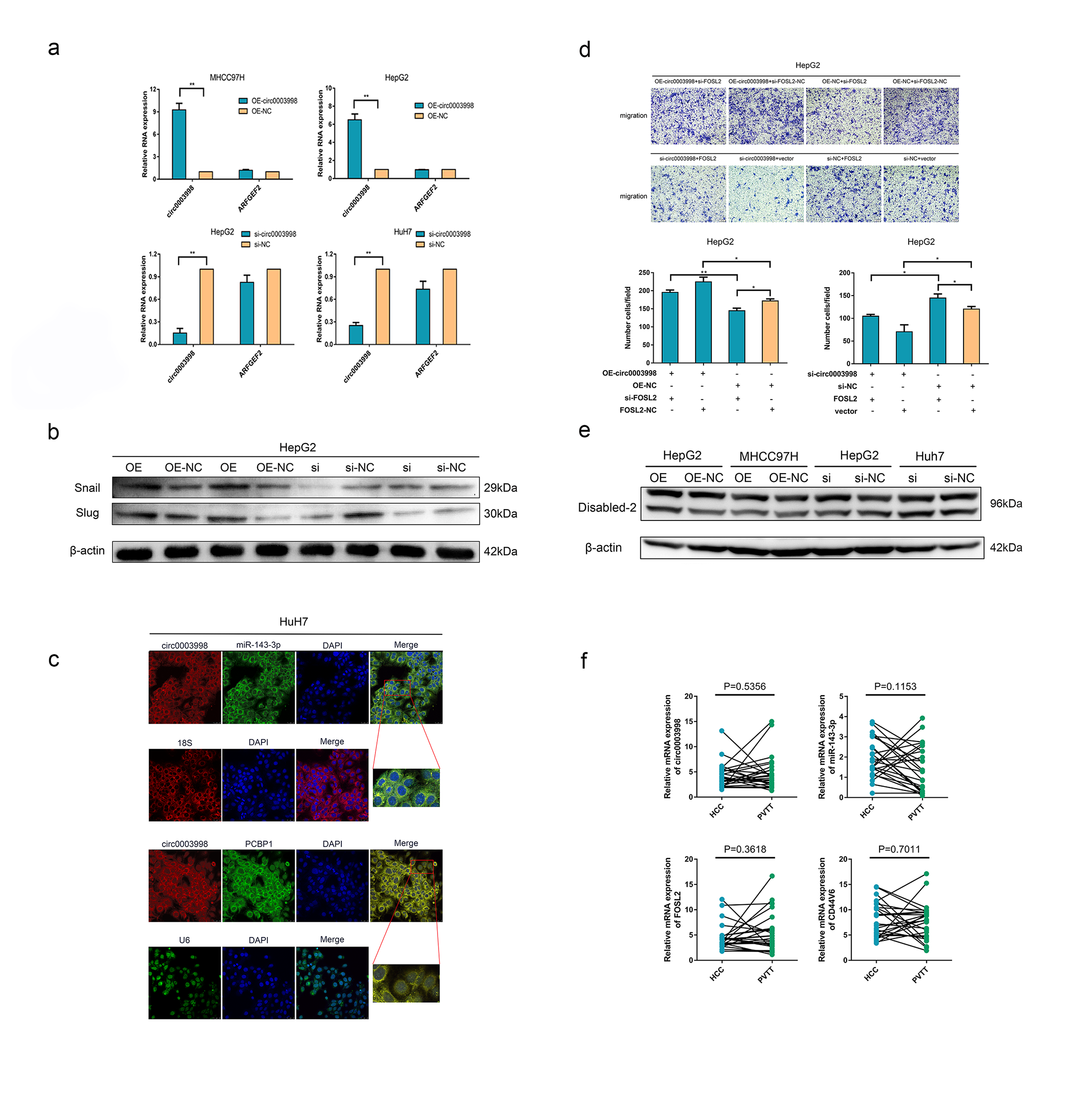

Supplement: Supplementary file 7 — Additional file 7: Fig. S1 (a) QRT-PCR detected the relative expression level of circ0003998 after over expressing or silencing circ0003998 in HCC cells. (b) The relative protein expression level of Snail and Slug in the HCC cells with circ0003998 over expression and silencing. (c) FISH was performed to observe the cellular location of circ0003998 (red), miR-143-3p (green) and PCBP1 (green) in HuH7 cells (magnification, × 400, scale bar, 25 μm). (d) Rescue transwell assay was performed after trans-fection with indicated vectors, OE-FOSL2 or si-FOSL2 (magnification, × 100, scale bar, 100 μm). (e) The relative protein expression level of DAB2 in HCC cells with circ0003998 over expression and silencing. (f) The relative mRNA expression of circ003998, FOSL2 and CD44v6 in HCC and PVTT tissues. *p-value< 0.05, **p-value< 0.01, ***p-value< 0.001. [file 13046_2020_1576_MOESM7_ESM.tif]
