# Supplementary material for: Hsa_circ_0003998 promotes epithelial to mesenchymal transition of hepatocellular carcinoma by sponging miR-143-3p and PCBP1
Source: J Exp Clin Cancer Res. 2020 Jun 17;39:114. doi: 10.1186/s13046-020-01576-0 (PMC7302140; doi:10.1186/s13046-020-01576-0)
Supplement: Supplementary file 3 — Additional file 3. Predicted target genes for hsa-miR-143-3p in TargetScan and miRanda. [file 13046_2020_1576_MOESM3_ESM.pdf]

Predicted target genes for hsa-miR-143-3p

# Column A: Seqname, the name the sequence.
# Column B: GeneSymbol, the official gene symbol of the sequence.
# Column C: Type, the type of the transcipt.
# Column D: Diagrams, the diagrams illustrate key features for miRNA binding.
# Column E: Total, the total number of the binding sites on the targets.
# Column F: Context+, the sum of the context+ scores used in TargetScan after version 6.0. More negative is better.
# Column G: Context, the sum of the context scores used in TargetScan before version 5.x. More negative is better.
# Column H: Structure, the sum of the structure scores used in miRanda. The higher the better.
# Column I: Energy, the sum of the free energy predicted by miRanda. More negative is better.
# Column J: Branch Length, the sum of the branch length in the phylogenetic tree of different species. The higher means the binding site is more conserved.
# Column K: Pct, the probability of preferentially conserved targeting. This score reflected the Bayesian estimate of the probability that a site is conserved due to selective maintenance of miRNA targeting rather than by chance or any other reason not pertinent to miRNA targeting.
# Column L ~ Column W: Counting numbers for different seed match types.

Target genes for hsa-miR-143-3p

Target			TargetScan		miRanda		Conservation		Conserved Sites						Poorly Conserved Sites						Annotations
Seqname	GeneSymbol	Type	Context+	Context	Structure	Energy	Branch Le	Pct	8mer	7mer-m8	7mer-A1	6mer	Offset 6m	Imperfect	8mer	7mer-m8	7mer-A1	6mer	Offset 6m	Imperfect	UTR Lengt
NM_0010129	DISC1	Coding	-0.7	-0.833	300	-36.72	0	0.012	0	0	0	0	0	0	2	0	0	0	0	0	4442
NM_0011645	DISC1	Coding	-0.7	-0.833	300	-36.72	0	0.012	0	0	0	0	0	0	2	0	0	0	0	0	4442
NM_0011645	DISC1	Coding	-0.7	-0.833	300	-36.72	0	0.012	0	0	0	0	0	0	2	0	0	0	0	0	4442
NM_018662	DISC1	Coding	-0.7	-0.833	300	-36.72	0	0.012	0	0	0	0	0	0	2	0	0	0	0	0	4442
NM_014909	VASH1	Coding	-0.596	-0.706	301	-36.09	0	0.012	0	0	0	0	0	0	2	0	0	0	0	0	4002
NM_000370	TTPA	Coding	-0.529	-0.79	286	-29.46	0	0.012	0	0	0	0	0	0	2	0	0	0	0	0	1763
NM_005358	LMO7	Coding	-0.51	-0.659	288	-30.25	0	0.115	0	0	0	0	0	0	1	1	0	0	0	0	1927
NM_0013060	LMO7	Coding	-0.51	-0.659	288	-30.25	0	0.115	0	0	0	0	0	0	1	1	0	0	0	0	1927
NM_015842	LMO7	Coding	-0.51	-0.659	288	-30.25	0	0.115	0	0	0	0	0	0	1	1	0	0	0	0	1857
NM_0011952	DENND1B	Coding	-0.5	-0.785	317	-36.46	0	0.115	0	0	0	0	0	0	1	1	0	0	0	0	5711
NM_170721	MSI2	Coding	-0.477	-0.566	304	-33.62	0	0.115	0	0	0	0	0	0	1	1	0	0	0	0	1352
NM_0013222	MSI2	Coding	-0.477	-0.566	304	-33.62	0	0.115	0	0	0	0	0	0	1	1	0	0	0	0	1352
NM_0011601	HRH4	Coding	-0.467	-0.604	295	-33.46	0	0.115	0	0	0	0	0	0	1	1	0	0	0	0	3200
NM_0011682	ABL2	Coding	-0.428	-0.482	163	-20.47	0	0.006	0	0	0	0	0	0	1	0	0	0	0	0	8391
NM_0011682	ABL2	Coding	-0.428	-0.482	163	-20.47	0	0.006	0	0	0	0	0	0	1	0	0	0	0	0	8391
NM_0011682	ABL2	Coding	-0.428	-0.482	163	-20.47	0	0.006	0	0	0	0	0	0	1	0	0	0	0	0	8391
NM_007314	ABL2	Coding	-0.428	-0.482	163	-20.47	0	0.006	0	0	0	0	0	0	1	0	0	0	0	0	8391
NM_0011360	ABL2	Coding	-0.428	-0.482	163	-20.47	0	0.006	0	0	0	0	0	0	1	0	0	0	0	0	8391
NM_005158	ABL2	Coding	-0.428	-0.482	163	-20.47	0	0.006	0	0	0	0	0	0	1	0	0	0	0	0	8391
NM_0011682	ABL2	Coding	-0.428	-0.482	163	-20.47	0	0.006	0	0	0	0	0	0	1	0	0	0	0	0	8391
NM_021999	ITM2B	Coding	-0.414	-0.524	149	-16.47	0	0.006	0	0	0	0	0	0	1	0	0	0	0	0	872
NM_0013139	EIF2AK3	Coding	-0.405	-0.463	154	-18.42	0	0.006	0	0	0	0	0	0	1	0	0	0	0	0	996
NM_004836	EIF2AK3	Coding	-0.405	-0.463	154	-18.42	0	0.006	0	0	0	0	0	0	1	0	0	0	0	0	996
NM_0010776	UBXN2B	Coding	-0.405	-0.536	141	-11.75	0	0.006	0	0	0	0	0	0	1	0	0	0	0	0	3950
NM_016486	TMEM69	Coding	-0.403	-0.502	151	-18.23	0	0.006	0	0	0	0	0	0	1	0	0	0	0	0	531
NM_0011661	EME1	Coding	-0.4	-0.47	169	-22.28	0	0.006	0	0	0	0	0	0	1	0	0	0	0	0	520
NM_152463	EME1	Coding	-0.4	-0.47	169	-22.28	0	0.006	0	0	0	0	0	0	1	0	0	0	0	0	520
NM_016548	GOLM1	Coding	-0.395	-0.494	148	-13.71	0	0.006	0	0	0	0	0	0	1	0	0	0	0	0	1674
NM_177937	GOLM1	Coding	-0.395	-0.494	148	-13.71	0	0.006	0	0	0	0	0	0	1	0	0	0	0	0	1674
NM_052882	ZIM3	Coding	-0.39	-0.517	148	-17.75	0	0.006	0	0	0	0	0	0	1	0	0	0	0	0	822
NM_0012976	C1RL	Coding	-0.387	-0.452	142	-16.83	0	0.006	0	0	0	0	0	0	1	0	0	0	0	0	218
NM_002010	FGF9	Coding	-0.387	-0.506	154	-14.69	0	0.006	0	0	0	0	0	0	1	0	0	0	0	0	3066
NM_004281	BAG3	Coding	-0.383	-0.504	152	-18.11	0	0.006	0	0	0	0	0	0	1	0	0	0	0	0	535
NM_214711	PRR27	Coding	-0.38	-0.455	156	-17.15	0	0.006	0	0	0	0	0	0	1	0	0	0	0	0	3948
NM_0010109	ATP11C	Coding	-0.379	-0.449	148	-18.51	0	0.006	0	0	0	0	0	0	1	0	0	0	0	0	2554
NM_004768	SRSF11	Coding	-0.378	-0.458	163	-18.3	0	0.006	0	0	0	0	0	0	1	0	0	0	0	0	1213
NM_0011909	SRSF11	Coding	-0.378	-0.458	163	-18.3	0	0.006	0	0	0	0	0	0	1	0	0	0	0	0	1213
NM_003676	DEGS1	Coding	-0.378	-0.524	150	-15.09	0	0.006	0	0	0	0	0	0	1	0	0	0	0	0	962
NM_0013215	DEGS1	Coding	-0.378	-0.524	150	-15.09	0	0.006	0	0	0	0	0	0	1	0	0	0	0	0	962
NM_0013215	DEGS1	Coding	-0.378	-0.524	150	-15.09	0	0.006	0	0	0	0	0	0	1	0	0	0	0	0	1132
NM_152653	UBE2E2	Coding	-0.377	-0.493	151	-17.28	0	0.006	0	0	0	0	0	0	1	0	0	0	0	0	974

NM_0012785	IL36G	Coding	-0.376	-0.465	157	-20.64	0	0.006	0	0	0	0	0	0	1	0	0	0	0	0	623
NM_019618	IL36G	Coding	-0.376	-0.465	157	-20.64	0	0.006	0	0	0	0	0	0	1	0	0	0	0	0	623
NM_052866	ADAMTSL1	Coding	-0.376	-0.432	155	-19.34	0	0.006	0	0	0	0	0	0	1	0	0	0	0	0	150
NM_000259	MYO5A	Coding	-0.376	-0.523	147	-16.84	0	0.006	0	0	0	0	0	0	1	0	0	0	0	0	6413
NM_0011424	MYO5A	Coding	-0.376	-0.523	147	-16.84	0	0.006	0	0	0	0	0	0	1	0	0	0	0	0	6413
NM_004329	BMPR1A	Coding	-0.37	-0.509	149	-14.72	0	0.006	0	0	0	0	0	0	1	0	0	0	0	0	1469
NM_0012426	AHCYL1	Coding	-0.37	-0.498	148	-14.71	0	0.006	0	0	0	0	0	0	1	0	0	0	0	0	2062
NM_006621	AHCYL1	Coding	-0.37	-0.498	148	-14.71	0	0.006	0	0	0	0	0	0	1	0	0	0	0	0	2062
NM_0012426	AHCYL1	Coding	-0.37	-0.498	148	-14.71	0	0.006	0	0	0	0	0	0	1	0	0	0	0	0	2062
NM_0012426	AHCYL1	Coding	-0.37	-0.498	148	-14.71	0	0.006	0	0	0	0	0	0	1	0	0	0	0	0	2062
NM_0012426	AHCYL1	Coding	-0.37	-0.498	148	-14.71	0	0.006	0	0	0	0	0	0	1	0	0	0	0	0	2062
NM_152389	CFAP65	Coding	-0.369	-0.462	145	-13.95	0	0.006	0	0	0	0	0	0	1	0	0	0	0	0	1640
NM_014366	GNL3	Coding	-0.369	-0.48	145	-14.05	0	0.006	0	0	0	0	0	0	1	0	0	0	0	0	219
NM_206825	GNL3	Coding	-0.369	-0.48	145	-14.05	0	0.006	0	0	0	0	0	0	1	0	0	0	0	0	219
NM_206826	GNL3	Coding	-0.369	-0.48	145	-14.05	0	0.006	0	0	0	0	0	0	1	0	0	0	0	0	219
NM_024490	ATP10A	Coding	-0.368	-0.524	155	-15.8	0	0.006	0	0	0	0	0	0	1	0	0	0	0	0	628
NM_005253	FOSL2	Coding	-0.368	-0.397	152	-17.79	0	0.006	0	0	0	0	0	0	1	0	0	0	0	0	2201
NM_0012821	FTSJ1	Coding	-0.366	-0.455	142	-11.75	0	0.006	0	0	0	0	0	0	1	0	0	0	0	0	638
NM_012280	FTSJ1	Coding	-0.366	-0.455	142	-11.75	0	0.006	0	0	0	0	0	0	1	0	0	0	0	0	638
NM_177439	FTSJ1	Coding	-0.366	-0.455	142	-11.75	0	0.006	0	0	0	0	0	0	1	0	0	0	0	0	638
NM_032849	MEDAG	Coding	-0.363	-0.449	159	-16.59	0	0.006	0	0	0	0	0	0	1	0	0	0	0	0	1137
NM_0012919	WASF3	Coding	-0.362	-0.475	149	-14.93	0	0.006	0	0	0	0	0	0	1	0	0	0	0	0	3100
NM_006646	WASF3	Coding	-0.362	-0.475	149	-14.93	0	0.006	0	0	0	0	0	0	1	0	0	0	0	0	3100
NM_173694	ATP11C	Coding	-0.362	-0.438	148	-18.51	0	0.006	0	0	0	0	0	0	1	0	0	0	0	0	2620
NM_173602	DIP2B	Coding	-0.36	-0.411	161	-23.64	0	0.006	0	0	0	0	0	0	1	0	0	0	0	0	3828
NM_005290	GPR15	Coding	-0.36	-0.488	146	-14.44	0	0.006	0	0	0	0	0	0	1	0	0	0	0	0	1563
NM_0011934	SECISBP2L	Coding	-0.359	-0.425	162	-20.5	0	0.006	0	0	0	0	0	0	1	0	0	0	0	0	3606
NM_014701	SECISBP2L	Coding	-0.359	-0.425	162	-20.5	0	0.006	0	0	0	0	0	0	1	0	0	0	0	0	3606
NM_152599	MFSD6L	Coding	-0.358	-0.39	146	-17.17	0	0.006	0	0	0	0	0	0	1	0	0	0	0	0	250
NM_0012572	ZNF451	Coding	-0.358	-0.416	143	-21.43	0	0.006	0	0	0	0	0	0	1	0	0	0	0	0	7618
NM_0011956	MLLT10	Coding	-0.357	-0.472	144	-13.44	0	0.006	0	0	0	0	0	0	1	0	0	0	0	0	243
NM_0011956	MLLT10	Coding	-0.357	-0.472	144	-13.44	0	0.006	0	0	0	0	0	0	1	0	0	0	0	0	243
NM_0011956	MLLT10	Coding	-0.357	-0.472	144	-13.44	0	0.006	0	0	0	0	0	0	1	0	0	0	0	0	508
NM_175854	PAN3	Coding	-0.356	-0.437	150	-16.24	0	0.006	0	0	0	0	0	0	1	0	0	0	0	0	2816
NM_0012817	MLIP	Coding	-0.355	-0.48	145	-15.28	0	0.006	0	0	0	0	0	0	1	0	0	0	0	0	2882
NM_0012564	DENND2C	Coding	-0.354	-0.446	145	-14.15	0	0.006	0	0	0	0	0	0	1	0	0	0	0	0	1025
NM_198459	DENND2C	Coding	-0.354	-0.446	145	-14.15	0	0.006	0	0	0	0	0	0	1	0	0	0	0	0	1025
NM_145111	FAM200A	Coding	-0.354	-0.451	157	-23.74	0	0.006	0	0	0	0	0	0	1	0	0	0	0	0	386
NM_000189	HK2	Coding	-0.354	-0.414	152	-16.54	0	0.006	0	0	0	0	0	0	1	0	0	0	0	0	2413
NM_0011127	VAX1	Coding	-0.351	-0.433	155	-19.92	0	0.006	0	0	0	0	0	0	1	0	0	0	0	0	718
NM_0011847	GYG2	Coding	-0.349	-0.421	154	-15.57	0	0.006	0	0	0	0	0	0	1	0	0	0	0	0	1607
NM_0011847	GYG2	Coding	-0.349	-0.421	154	-15.57	0	0.006	0	0	0	0	0	0	1	0	0	0	0	0	1607
NM_0011847	GYG2	Coding	-0.349	-0.421	154	-15.57	0	0.006	0	0	0	0	0	0	1	0	0	0	0	0	1607
NM_003918	GYG2	Coding	-0.349	-0.421	154	-15.57	0	0.006	0	0	0	0	0	0	1	0	0	0	0	0	1607
NM_0010798	GYG2	Coding	-0.349	-0.421	154	-15.57	0	0.006	0	0	0	0	0	0	1	0	0	0	0	0	1607
NM_017433	MYO3A	Coding	-0.349	-0.439	153	-16.05	0	0.006	0	0	0	0	0	0	1	0	0	0	0	0	573
NM_0011597	SLC39A11	Coding	-0.349	-0.449	152	-16.08	0	0.006	0	0	0	0	0	0	1	0	0	0	0	0	1638
NM_139177	SLC39A11	Coding	-0.349	-0.449	152	-16.08	0	0.006	0	0	0	0	0	0	1	0	0	0	0	0	1638
NM_016001	UTP18	Coding	-0.348	-0.463	141	-13.23	0	0.006	0	0	0	0	0	0	1	0	0	0	0	0	164
NM_016540	GPR83	Coding	-0.348	-0.429	155	-16.04	0	0.006	0	0	0	0	0	0	1	0	0	0	0	0	2838
NM_014252	SLC25A15	Coding	-0.348	-0.348	158	-22.44	0	0.006	0	0	0	0	0	0	1	0	0	0	0	0	2793
NM_0012424	LOC389831	Coding	-0.347	-0.427	156	-17.56	0	0.006	0	0	0	0	0	0	1	0	0	0	0	0	1786
NM_0013220	LOC102724951	Coding	-0.347	-0.427	156	-17.56	0	0.006	0	0	0	0	0	0	1	0	0	0	0	0	1786
NM_0013220	LOC102724219	Coding	-0.347	-0.427	156	-17.56	0	0.006	0	0	0	0	0	0	1	0	0	0	0	0	1786
NM_0013220	LOC102724843	Coding	-0.347	-0.427	156	-17.56	0	0.006	0	0	0	0	0	0	1	0	0	0	0	0	1786
NM_000599	IGFBP5	Coding	-0.344	-0.45	146	-16.06	0	0.006	0	0	0	0	0	0	1	0	0	0	0	0	4646
NM_0011438	COPS2	Coding	-0.343	-0.448	157	-17.1	0	0.006	0	0	0	0	0	0	1	0	0	0	0	0	2676
NM_004236	COPS2	Coding	-0.343	-0.448	157	-17.1	0	0.006	0	0	0	0	0	0	1	0	0	0	0	0	2676
NM_152890	COL24A1	Coding	-0.342	-0.475	145	-13.45	0	0.006	0	0	0	0	0	0	1	0	0	0	0	0	1313
NM_002173	IFNA16	Coding	-0.34	-0.434	147	-12.56	0	0.006	0	0	0	0	0	0	1	0	0	0	0	0	363

NM_0013044	PTPN23	Coding	-0.34	-0.348	149	-17.35	0	0.006	0	0	0	0	0	0	1	0	0	0	0	0	256
NM_015466	PTPN23	Coding	-0.34	-0.348	149	-17.35	0	0.006	0	0	0	0	0	0	1	0	0	0	0	0	256
NM_014805	EPM2AIP1	Coding	-0.339	-0.458	148	-15.74	0	0.006	0	0	0	0	0	0	1	0	0	0	0	0	5388
NM_030625	TET1	Coding	-0.338	-0.4	156	-17.86	0	0.006	0	0	0	0	0	0	1	0	0	0	0	0	2668
NM_0011662	FHIT	Coding	-0.338	-0.373	164	-22.79	0	0.006	0	0	0	0	0	0	1	0	0	0	0	0	2296
NM_002172	IFNA14	Coding	-0.336	-0.428	147	-13.26	0	0.006	0	0	0	0	0	0	1	0	0	0	0	0	164
NM_0010037	RECQL5	Coding	-0.336	-0.418	153	-17.98	0	0.006	0	0	0	0	0	0	1	0	0	0	0	0	926
NM_018452	TMEM242	Coding	-0.335	-0.48	159	-19.28	0	0.006	0	0	0	0	0	0	1	0	0	0	0	0	3879
NM_0011706	SCUBE2	Coding	-0.335	-0.435	153	-17.98	0	0.006	0	0	0	0	0	0	1	0	0	0	0	0	1545
NM_020974	SCUBE2	Coding	-0.335	-0.435	153	-17.98	0	0.006	0	0	0	0	0	0	1	0	0	0	0	0	1545
NM_002012	FHIT	Coding	-0.335	-0.371	164	-22.79	0	0.006	0	0	0	0	0	0	1	0	0	0	0	0	2307
NM_001047	SRD5A1	Coding	-0.334	-0.431	144	-16.49	0	0.006	0	0	0	0	0	0	1	0	0	0	0	0	1294
NM_0012043	NPR3	Coding	-0.333	-0.397	157	-19.61	0	0.006	0	0	0	0	0	0	1	0	0	0	0	0	5379
NM_000908	NPR3	Coding	-0.333	-0.397	157	-19.61	0	0.006	0	0	0	0	0	0	1	0	0	0	0	0	5379
NM_0012043	NPR3	Coding	-0.333	-0.397	157	-19.61	0	0.006	0	0	0	0	0	0	1	0	0	0	0	0	5379
NM_001549	IFIT3	Coding	-0.331	-0.364	154	-20.14	0	0.006	0	0	0	0	0	0	1	0	0	0	0	0	840
NM_0010316	IFIT3	Coding	-0.331	-0.364	154	-20.14	0	0.006	0	0	0	0	0	0	1	0	0	0	0	0	840
NM_0012897	IFIT3	Coding	-0.331	-0.364	154	-20.14	0	0.006	0	0	0	0	0	0	1	0	0	0	0	0	840
NM_0012897	IFIT3	Coding	-0.331	-0.364	154	-20.14	0	0.006	0	0	0	0	0	0	1	0	0	0	0	0	840
NM_153809	TAF1L	Coding	-0.33	-0.421	148	-16.02	0	0.006	0	0	0	0	0	0	1	0	0	0	0	0	645
NM_0011272	CACNA1A	Coding	-0.33	-0.458	149	-14.42	0	0.006	0	0	0	0	0	0	1	0	0	0	0	0	871
NM_023035	CACNA1A	Coding	-0.33	-0.458	149	-14.42	0	0.006	0	0	0	0	0	0	1	0	0	0	0	0	871
NM_017523	XAF1	Coding	-0.329	-0.457	143	-13.9	0	0.006	0	0	0	0	0	0	1	0	0	0	0	0	2476
NM_199139	XAF1	Coding	-0.329	-0.457	143	-13.9	0	0.006	0	0	0	0	0	0	1	0	0	0	0	0	2476
NM_213568	SLC39A3	Coding	-0.329	-0.37	156	-21.94	0	0.006	0	0	0	0	0	0	1	0	0	0	0	0	2414
NM_0011422	ARFGAP3	Coding	-0.329	-0.453	145	-11.75	0	0.006	0	0	0	0	0	0	1	0	0	0	0	0	1062
NM_014570	ARFGAP3	Coding	-0.329	-0.453	145	-11.75	0	0.006	0	0	0	0	0	0	1	0	0	0	0	0	1062
NM_0011310	ATG10	Coding	-0.328	-0.428	153	-13.99	0	0.006	0	0	0	0	0	0	1	0	0	0	0	0	1334
NM_031482	ATG10	Coding	-0.328	-0.428	153	-13.99	0	0.006	0	0	0	0	0	0	1	0	0	0	0	0	1334
NM_0012520	PCMT1	Coding	-0.328	-0.316	156	-21.69	0	0.006	0	0	0	0	0	0	1	0	0	0	0	0	793
NM_0012520	PCMT1	Coding	-0.328	-0.316	156	-21.69	0	0.006	0	0	0	0	0	0	1	0	0	0	0	0	793
NM_021057	IFNA7	Coding	-0.327	-0.4	155	-18.07	0	0.006	0	0	0	0	0	0	1	0	0	0	0	0	127
NM_0011437	ASAP3	Coding	-0.326	-0.412	146	-15.13	0	0.006	0	0	0	0	0	0	1	0	0	0	0	0	1293
NM_017707	ASAP3	Coding	-0.326	-0.412	146	-15.13	0	0.006	0	0	0	0	0	0	1	0	0	0	0	0	1293
NM_152485	C1orf74	Coding	-0.326	-0.431	140	-12.38	0	0.006	0	0	0	0	0	0	1	0	0	0	0	0	3617
NM_000068	CACNA1A	Coding	-0.325	-0.455	149	-14.42	0	0.006	0	0	0	0	0	0	1	0	0	0	0	0	1604
NM_0011272	CACNA1A	Coding	-0.325	-0.455	149	-14.42	0	0.006	0	0	0	0	0	0	1	0	0	0	0	0	1604
NM_0011740	CACNA1A	Coding	-0.325	-0.455	149	-14.42	0	0.006	0	0	0	0	0	0	1	0	0	0	0	0	1604
NM_032862	TIGD5	Coding	-0.325	-0.33	154	-21.33	0	0.006	0	0	0	0	0	0	1	0	0	0	0	0	483
NM_0013222	MSI2	Coding	-0.324	-0.353	158	-18.03	0	0.006	0	0	0	0	0	0	1	0	0	0	0	0	5172
NM_138962	MSI2	Coding	-0.324	-0.353	158	-18.03	0	0.006	0	0	0	0	0	0	1	0	0	0	0	0	5172
NM_133334	WHSC1	Coding	-0.323	-0.443	145	-12.89	0	0.006	0	0	0	0	0	0	1	0	0	0	0	0	6417
NM_0013009	ZNF570	Coding	-0.322	-0.413	152	-14.51	0	0.006	0	0	0	0	0	0	1	0	0	0	0	0	3419
NM_0013219	ZNF570	Coding	-0.322	-0.413	152	-14.51	0	0.006	0	0	0	0	0	0	1	0	0	0	0	0	3419
NM_0013219	ZNF570	Coding	-0.322	-0.413	152	-14.51	0	0.006	0	0	0	0	0	0	1	0	0	0	0	0	3419
NM_0013219	ZNF570	Coding	-0.322	-0.413	152	-14.51	0	0.006	0	0	0	0	0	0	1	0	0	0	0	0	3419
NM_144694	ZNF570	Coding	-0.322	-0.413	152	-14.51	0	0.006	0	0	0	0	0	0	1	0	0	0	0	0	3419
NM_0013219	ZNF570	Coding	-0.322	-0.413	152	-14.51	0	0.006	0	0	0	0	0	0	1	0	0	0	0	0	3419
NM_207435	C12orf76	Coding	-0.322	-0.396	149	-17.16	0	0.006	0	0	0	0	0	0	1	0	0	0	0	0	1174
NM_014614	PSME4	Coding	-0.32	-0.448	152	-14.48	0	0.006	0	0	0	0	0	0	1	0	0	0	0	0	1511
NM_006578	GNB5	Coding	-0.32	-0.415	144	-11.75	0	0.006	0	0	0	0	0	0	1	0	0	0	0	0	1831
NM_016194	GNB5	Coding	-0.32	-0.415	144	-11.75	0	0.006	0	0	0	0	0	0	1	0	0	0	0	0	1831
NM_022140	EPB41L4A	Coding	-0.319	-0.365	146	-15.57	0	0.006	0	0	0	0	0	0	1	0	0	0	0	0	2372
NM_145315	LACE1	Coding	-0.318	-0.456	140	-11.75	0	0.006	0	0	0	0	0	0	1	0	0	0	0	0	3359
NM_0010070	ZNF182	Coding	-0.318	-0.417	146	-13.6	0	0.006	0	0	0	0	0	0	1	0	0	0	0	0	1316
NM_006962	ZNF182	Coding	-0.318	-0.417	146	-13.6	0	0.006	0	0	0	0	0	0	1	0	0	0	0	0	1316
NM_0011780	ZNF182	Coding	-0.318	-0.417	146	-13.6	0	0.006	0	0	0	0	0	0	1	0	0	0	0	0	1316
NM_032228	FAR1	Coding	-0.316	-0.418	140	-11.77	0	0.006	0	0	0	0	0	0	1	0	0	0	0	0	3572
NM_0012520	PCMT1	Coding	-0.315	-0.307	156	-21.69	0	0.006	0	0	0	0	0	0	1	0	0	0	0	0	843
NM_0012520	PCMT1	Coding	-0.315	-0.307	156	-21.69	0	0.006	0	0	0	0	0	0	1	0	0	0	0	0	843

NM_005389	PCMT1	Coding	-0.315	-0.307	156	-21.69	0	0.006	0	0	0	0	0	0	1	0	0	0	0	0	843
NM_003454	ZNF200	Coding	-0.315	-0.42	140	-14.08	0	0.006	0	0	0	0	0	0	1	0	0	0	0	0	1567
NM_0011454	ZNF200	Coding	-0.315	-0.42	140	-14.08	0	0.006	0	0	0	0	0	0	1	0	0	0	0	0	1567
NM_198088	ZNF200	Coding	-0.315	-0.42	140	-14.08	0	0.006	0	0	0	0	0	0	1	0	0	0	0	0	1567
NM_0011454	ZNF200	Coding	-0.315	-0.42	140	-14.08	0	0.006	0	0	0	0	0	0	1	0	0	0	0	0	1567
NM_198087	ZNF200	Coding	-0.315	-0.42	140	-14.08	0	0.006	0	0	0	0	0	0	1	0	0	0	0	0	1567
NM_0011454	ZNF200	Coding	-0.315	-0.42	140	-14.08	0	0.006	0	0	0	0	0	0	1	0	0	0	0	0	1567
NM_207372	SH2D4B	Coding	-0.314	-0.372	150	-17.1	0	0.006	0	0	0	0	0	0	1	0	0	0	0	0	2479
NM_0011457	SH2D4B	Coding	-0.314	-0.372	150	-17.1	0	0.006	0	0	0	0	0	0	1	0	0	0	0	0	2479
NM_015407	ABHD14A	Coding	-0.313	-0.393	146	-15.34	0	0.006	0	0	0	0	0	0	1	0	0	0	0	0	182
NM_0011031	GIGYF2	Coding	-0.313	-0.324	143	-14.66	0	0.006	0	0	0	0	0	0	1	0	0	0	0	0	3719
NM_0011031	GIGYF2	Coding	-0.313	-0.324	143	-14.66	0	0.006	0	0	0	0	0	0	1	0	0	0	0	0	3719
NM_0011031	GIGYF2	Coding	-0.313	-0.324	143	-14.66	0	0.006	0	0	0	0	0	0	1	0	0	0	0	0	3719
NM_015575	GIGYF2	Coding	-0.313	-0.324	143	-14.66	0	0.006	0	0	0	0	0	0	1	0	0	0	0	0	3719
NM_0011450	MEIOC	Coding	-0.312	-0.391	145	-13.27	0	0.006	0	0	0	0	0	0	1	0	0	0	0	0	1601
NM_0012564	PDLIM5	Coding	-0.31	-0.428	162	-19.45	0	0.006	0	0	0	0	0	0	1	0	0	0	0	0	631
NM_174977	SEC14L4	Coding	-0.31	-0.376	147	-14.13	0	0.006	0	0	0	0	0	0	1	0	0	0	0	0	1196
NM_001872	CPB2	Coding	-0.308	-0.37	145	-13.85	0	0.006	0	0	0	0	0	0	1	0	0	0	0	0	427
NM_0012788	CPB2	Coding	-0.308	-0.37	145	-13.85	0	0.006	0	0	0	0	0	0	1	0	0	0	0	0	427
NM_144628	TBC1D20	Coding	-0.308	-0.364	154	-16.67	0	0.006	0	0	0	0	0	0	1	0	0	0	0	0	3109
NM_0011598	ZNF583	Coding	-0.308	-0.398	140	-13.71	0	0.006	0	0	0	0	0	0	1	0	0	0	0	0	663
NM_152478	ZNF583	Coding	-0.308	-0.398	140	-13.71	0	0.006	0	0	0	0	0	0	1	0	0	0	0	0	663
NM_0011598	ZNF583	Coding	-0.308	-0.398	140	-13.71	0	0.006	0	0	0	0	0	0	1	0	0	0	0	0	663
NM_001690	ATP6V1A	Coding	-0.306	-0.388	148	-13.3	0	0.006	0	0	0	0	0	0	1	0	0	0	0	0	2631
NM_0011454	CCDC110	Coding	-0.304	-0.401	140	-13.02	0	0.006	0	0	0	0	0	0	1	0	0	0	0	0	318
NM_152775	CCDC110	Coding	-0.304	-0.401	140	-13.02	0	0.006	0	0	0	0	0	0	1	0	0	0	0	0	318
NM_018268	WDR41	Coding	-0.304	-0.365	158	-17.39	0	0.006	0	0	0	0	0	0	1	0	0	0	0	0	2202
NM_0013007	RASGEF1B	Coding	-0.302	-0.338	165	-21.42	0	0.006	0	0	0	0	0	0	1	0	0	0	0	0	1375
NM_0013007	RASGEF1B	Coding	-0.302	-0.338	165	-21.42	0	0.006	0	0	0	0	0	0	1	0	0	0	0	0	1375
NM_152545	RASGEF1B	Coding	-0.302	-0.338	165	-21.42	0	0.006	0	0	0	0	0	0	1	0	0	0	0	0	1375
NM_0010057	NUMB	Coding	-0.302	-0.361	150	-15.62	0	0.006	0	0	0	0	0	0	1	0	0	0	0	0	1368
NM_0010057	NUMB	Coding	-0.302	-0.361	150	-15.62	0	0.006	0	0	0	0	0	0	1	0	0	0	0	0	1368
NM_0010057	NUMB	Coding	-0.302	-0.361	150	-15.62	0	0.006	0	0	0	0	0	0	1	0	0	0	0	0	1368
NM_0013201	NUMB	Coding	-0.302	-0.361	150	-15.62	0	0.006	0	0	0	0	0	0	1	0	0	0	0	0	1368
NM_003744	NUMB	Coding	-0.302	-0.361	150	-15.62	0	0.006	0	0	0	0	0	0	1	0	0	0	0	0	1368
NM_145238	ZSCAN20	Coding	-0.301	-0.419	148	-12.56	0	0.006	0	0	0	0	0	0	1	0	0	0	0	0	919
NM_000168	GLI3	Coding	-0.301	-0.359	141	-12.29	0	0.006	0	0	0	0	0	0	1	0	0	0	0	0	3380
NM_0010089	STK16	Coding	-0.301	-0.335	142	-17.69	0	0.006	0	0	0	0	0	0	1	0	0	0	0	0	1778
NM_080723	NRSN1	Coding	-0.3	-0.393	150	-15.67	0	0.006	0	0	0	0	0	0	1	0	0	0	0	0	1583
NM_007198	PROSC	Coding	-0.299	-0.44	150	-13.34	0	0.006	0	0	0	0	0	0	1	0	0	0	0	0	1664
NM_030788	DCSTAMP	Coding	-0.299	-0.367	149	-16.06	0	0.006	0	0	0	0	0	0	1	0	0	0	0	0	491
NM_198541	IGFL1	Coding	-0.298	-0.387	144	-13.06	0	0.006	0	0	0	0	0	0	1	0	0	0	0	0	413
NM_139032	MAPK7	Coding	-0.298	-0.3	158	-22.53	0	0.006	0	0	0	0	0	0	1	0	0	0	0	0	313
NM_139033	MAPK7	Coding	-0.298	-0.3	158	-22.53	0	0.006	0	0	0	0	0	0	1	0	0	0	0	0	313
NM_002749	MAPK7	Coding	-0.298	-0.3	158	-22.53	0	0.006	0	0	0	0	0	0	1	0	0	0	0	0	313
NM_139034	MAPK7	Coding	-0.298	-0.3	158	-22.53	0	0.006	0	0	0	0	0	0	1	0	0	0	0	0	313
NM_001165	BIRC3	Coding	-0.297	-0.388	146	-12.08	0	0.006	0	0	0	0	0	0	1	0	0	0	0	0	2302
NM_182962	BIRC3	Coding	-0.297	-0.388	146	-12.08	0	0.006	0	0	0	0	0	0	1	0	0	0	0	0	2302
NM_0010108	TECRL	Coding	-0.296	-0.413	145	-14.4	0	0.006	0	0	0	0	0	0	1	0	0	0	0	0	1613
NM_006930	SKP1	Coding	-0.295	-0.354	152	-18.64	0	0.006	0	0	0	0	0	0	1	0	0	0	0	0	2037
NM_170679	SKP1	Coding	-0.295	-0.354	152	-18.64	0	0.006	0	0	0	0	0	0	1	0	0	0	0	0	1342
NM_0011718	MCF2	Coding	-0.295	-0.373	146	-15.04	0	0.006	0	0	0	0	0	0	1	0	0	0	0	0	672
NM_0011718	MCF2	Coding	-0.295	-0.373	146	-15.04	0	0.006	0	0	0	0	0	0	1	0	0	0	0	0	672
NM_018036	ATG2B	Coding	-0.295	-0.333	160	-19.82	0	0.006	0	0	0	0	0	0	1	0	0	0	0	0	4497
NM_0012567	SSBP2	Coding	-0.295	-0.331	146	-14.59	0	0.006	0	0	0	0	0	0	1	0	0	0	0	0	3144
NM_0012567	SSBP2	Coding	-0.295	-0.331	146	-14.59	0	0.006	0	0	0	0	0	0	1	0	0	0	0	0	3144
NM_0012567	SSBP2	Coding	-0.295	-0.331	146	-14.59	0	0.006	0	0	0	0	0	0	1	0	0	0	0	0	3144
NM_0012567	SSBP2	Coding	-0.295	-0.331	146	-14.59	0	0.006	0	0	0	0	0	0	1	0	0	0	0	0	3144
NM_0012567	SSBP2	Coding	-0.295	-0.331	146	-14.59	0	0.006	0	0	0	0	0	0	1	0	0	0	0	0	3144
NM_012446	SSBP2	Coding	-0.295	-0.331	146	-14.59	0	0.006	0	0	0	0	0	0	1	0	0	0	0	0	3144

NM_000655	SELL	Coding	-0.295	-0.387	146	-14.22	0	0.006	0	0	0	0	0	0	1	0	0	0	0	0	1119
NM_004962	GDF10	Coding	-0.294	-0.394	149	-18.51	0	0.006	0	0	0	0	0	0	1	0	0	0	0	0	782
NM_022754	SFXN1	Coding	-0.293	-0.396	148	-16.46	0	0.006	0	0	0	0	0	0	1	0	0	0	0	0	3007
NM_004613	TGM2	Coding	-0.291	-0.364	152	-14.79	0	0.006	0	0	0	0	0	0	1	0	0	0	0	0	2832
NM_000900	MGP	Coding	-0.29	-0.419	146	-16.45	0	0.006	0	0	0	0	0	0	1	0	0	0	0	0	958
NM_0011908	MGP	Coding	-0.29	-0.419	146	-16.45	0	0.006	0	0	0	0	0	0	1	0	0	0	0	0	958
NM_0011904	CLSPN	Coding	-0.288	-0.437	147	-15.01	0	0.006	0	0	0	0	0	0	1	0	0	0	0	0	4384
NM_022111	CLSPN	Coding	-0.288	-0.437	147	-15.01	0	0.006	0	0	0	0	0	0	1	0	0	0	0	0	4384
NM_0013140	ZNF526	Coding	-0.288	-0.364	152	-20.31	0	0.006	0	0	0	0	0	0	1	0	0	0	0	0	637
NM_133444	ZNF526	Coding	-0.288	-0.364	152	-20.31	0	0.006	0	0	0	0	0	0	1	0	0	0	0	0	637
NM_0011718	MCF2	Coding	-0.288	-0.368	146	-15.04	0	0.006	0	0	0	0	0	0	1	0	0	0	0	0	700
NM_005369	MCF2	Coding	-0.288	-0.368	146	-15.04	0	0.006	0	0	0	0	0	0	1	0	0	0	0	0	700
NM_0010998	MCF2	Coding	-0.288	-0.368	146	-15.04	0	0.006	0	0	0	0	0	0	1	0	0	0	0	0	700
NM_0011718	MCF2	Coding	-0.288	-0.368	146	-15.04	0	0.006	0	0	0	0	0	0	1	0	0	0	0	0	700
NM_0013185	BEND6	Coding	-0.288	-0.297	159	-19.6	0	0.006	0	0	0	0	0	0	1	0	0	0	0	0	2743
NM_0011422	DHRS9	Coding	-0.287	-0.356	146	-12.13	0	0.11	0	0	0	0	0	0	0	1	0	0	0	0	400
NM_0012897	DHRS9	Coding	-0.287	-0.356	146	-12.13	0	0.11	0	0	0	0	0	0	0	1	0	0	0	0	400
NM_0011422	DHRS9	Coding	-0.287	-0.356	146	-12.13	0	0.11	0	0	0	0	0	0	0	1	0	0	0	0	400
NM_199204	DHRS9	Coding	-0.287	-0.356	146	-12.13	0	0.11	0	0	0	0	0	0	0	1	0	0	0	0	400
NM_0011461	MOGS	Coding	-0.287	-0.363	146	-14.1	0	0.006	0	0	0	0	0	0	1	0	0	0	0	0	218
NM_006302	MOGS	Coding	-0.287	-0.363	146	-14.1	0	0.006	0	0	0	0	0	0	1	0	0	0	0	0	218
NM_0013128	COX20	Coding	-0.286	-0.357	145	-10.47	0	0.11	0	0	0	0	0	0	0	1	0	0	0	0	1887
NM_198076	COX20	Coding	-0.286	-0.357	145	-10.47	0	0.11	0	0	0	0	0	0	0	1	0	0	0	0	1887
NM_0013128	COX20	Coding	-0.286	-0.357	145	-10.47	0	0.11	0	0	0	0	0	0	0	1	0	0	0	0	1887
NM_0013128	COX20	Coding	-0.286	-0.357	145	-10.47	0	0.11	0	0	0	0	0	0	0	1	0	0	0	0	1887
NM_0013128	COX20	Coding	-0.286	-0.357	145	-10.47	0	0.11	0	0	0	0	0	0	0	1	0	0	0	0	2054
NM_0013207	HTR2B	Coding	-0.286	-0.361	141	-9.62	0	0.11	0	0	0	0	0	0	0	1	0	0	0	0	284
NM_000867	HTR2B	Coding	-0.286	-0.361	141	-9.62	0	0.11	0	0	0	0	0	0	0	1	0	0	0	0	281
NM_0012573	DCSTAMP	Coding	-0.286	-0.358	149	-16.06	0	0.006	0	0	0	0	0	0	1	0	0	0	0	0	541
NM_0012703	ANKRD46	Coding	-0.285	-0.337	159	-15.02	0	0.006	0	0	0	0	0	0	1	0	0	0	0	0	825
NM_003188	MAP3K7	Coding	-0.285	-0.413	146	-12.44	0	0.006	0	0	0	0	0	0	1	0	0	0	0	0	2928
NM_145331	MAP3K7	Coding	-0.285	-0.413	146	-12.44	0	0.006	0	0	0	0	0	0	1	0	0	0	0	0	2928
NM_019859	HTR7	Coding	-0.285	-0.333	144	-15.26	0	0.006	0	0	0	0	0	0	1	0	0	0	0	0	1663
NM_015691	WWC3	Coding	-0.285	-0.368	153	-15.54	0	0.11	0	0	0	0	0	0	0	1	0	0	0	0	2977
NM_000872	HTR7	Coding	-0.284	-0.332	144	-15.26	0	0.006	0	0	0	0	0	0	1	0	0	0	0	0	1667
NM_0011848	PSG1	Coding	-0.282	-0.337	146	-18.68	0	0.006	0	0	0	0	0	0	1	0	0	0	0	0	673
NM_0011848	PSG1	Coding	-0.282	-0.337	146	-18.68	0	0.006	0	0	0	0	0	0	1	0	0	0	0	0	593
NM_001185	AZGP1	Coding	-0.281	-0.364	140	-11.75	0	0.006	0	0	0	0	0	0	1	0	0	0	0	0	276
NM_0013083	CCDC58	Coding	-0.281	-0.345	146	-14.38	0	0.006	0	0	0	0	0	0	1	0	0	0	0	0	278
NM_0010179	CCDC58	Coding	-0.281	-0.345	146	-14.38	0	0.006	0	0	0	0	0	0	1	0	0	0	0	0	278
NM_0012433	CCDC40	Coding	-0.281	-0.312	149	-22.54	0	0.006	0	0	0	0	0	0	1	0	0	0	0	0	422
NM_0010373	USP14	Coding	-0.281	-0.338	146	-14.91	0	0.006	0	0	0	0	0	0	1	0	0	0	0	0	2455
NM_005151	USP14	Coding	-0.281	-0.338	146	-14.91	0	0.006	0	0	0	0	0	0	1	0	0	0	0	0	2455
NM_000869	HTR3A	Coding	-0.281	-0.351	153	-15.95	0	0.006	0	0	0	0	0	0	1	0	0	0	0	0	549
NM_213621	HTR3A	Coding	-0.281	-0.351	153	-15.95	0	0.006	0	0	0	0	0	0	1	0	0	0	0	0	549
NM_0011617	HTR3A	Coding	-0.281	-0.351	153	-15.95	0	0.006	0	0	0	0	0	0	1	0	0	0	0	0	549
NM_0013079	UPRT	Coding	-0.281	-0.391	145	-13.83	0	0.006	0	0	0	0	0	0	1	0	0	0	0	0	1520
NM_145052	UPRT	Coding	-0.281	-0.391	145	-13.83	0	0.006	0	0	0	0	0	0	1	0	0	0	0	0	1386
NM_0010127	DUXA	Coding	-0.281	-0.412	161	-17.72	0	0.006	0	0	0	0	0	0	1	0	0	0	0	0	2686
NM_015513	CRELD1	Coding	-0.28	-0.394	150	-14.23	0	0.006	0	0	0	0	0	0	1	0	0	0	0	0	834
NM_0010317	CRELD1	Coding	-0.28	-0.394	150	-14.23	0	0.006	0	0	0	0	0	0	1	0	0	0	0	0	1022
NM_0010774	CRELD1	Coding	-0.28	-0.394	150	-14.23	0	0.006	0	0	0	0	0	0	1	0	0	0	0	0	834
NM_0011849	MITF	Coding	-0.28	-0.368	159	-21.21	0	0.006	0	0	0	0	0	0	1	0	0	0	0	0	699
NM_0012425	RIN2	Coding	-0.279	-0.368	146	-15.82	0	0.006	0	0	0	0	0	0	1	0	0	0	0	0	1523
NM_018993	RIN2	Coding	-0.279	-0.368	146	-15.82	0	0.006	0	0	0	0	0	0	1	0	0	0	0	0	1523
NM_004170	SLC1A1	Coding	-0.279	-0.341	141	-15.29	0	0.006	0	0	0	0	0	0	1	0	0	0	0	0	1911
NM_007375	TARDBP	Coding	-0.278	-0.377	154	-21.28	0	0.006	0	0	0	0	0	0	1	0	0	0	0	0	2838
NM_0011301	PSG8	Coding	-0.278	-0.331	146	-18.68	0	0.006	0	0	0	0	0	0	1	0	0	0	0	0	673
NM_0011301	PSG8	Coding	-0.278	-0.331	146	-18.68	0	0.006	0	0	0	0	0	0	1	0	0	0	0	0	673
NM_182548	LHFPL5	Coding	-0.278	-0.346	145	-10.47	0	0.11	0	0	0	0	0	0	0	1	0	0	0	0	1110

NM_023012	RSRC2	Coding	-0.277	-0.391	160	-18.93	0	0.11	0	0	0	0	0	0	0	1	0	0	0	0	884
NM_0012764	PSG4	Coding	-0.277	-0.329	146	-18.68	0	0.006	0	0	0	0	0	0	1	0	0	0	0	0	670
NM_0013163	PSG4	Coding	-0.277	-0.329	146	-18.68	0	0.006	0	0	0	0	0	0	1	0	0	0	0	0	670
NM_002780	PSG4	Coding	-0.277	-0.329	146	-18.68	0	0.006	0	0	0	0	0	0	1	0	0	0	0	0	670
NM_213633	PSG4	Coding	-0.277	-0.329	146	-18.68	0	0.006	0	0	0	0	0	0	1	0	0	0	0	0	670
NM_0012786	POLDIP3	Coding	-0.277	-0.36	161	-16.78	0	0.006	0	0	0	0	0	0	1	0	0	0	0	0	2070
NM_178136	POLDIP3	Coding	-0.277	-0.36	161	-16.78	0	0.006	0	0	0	0	0	0	1	0	0	0	0	0	2070
NM_032311	POLDIP3	Coding	-0.277	-0.36	161	-16.78	0	0.006	0	0	0	0	0	0	1	0	0	0	0	0	2070
NM_004500	HNRNPC	Coding	-0.276	-0.335	141	-9.57	0	0.11	0	0	0	0	0	0	0	1	0	0	0	0	2086
NM_0010774	HNRNPC	Coding	-0.276	-0.335	141	-9.57	0	0.11	0	0	0	0	0	0	0	1	0	0	0	0	2086
NM_031314	HNRNPC	Coding	-0.276	-0.335	141	-9.57	0	0.11	0	0	0	0	0	0	0	1	0	0	0	0	2086
NM_0010774	HNRNPC	Coding	-0.276	-0.335	141	-9.57	0	0.11	0	0	0	0	0	0	0	1	0	0	0	0	2086
NM_003341	UBE2E1	Coding	-0.276	-0.394	150	-15.06	0	0.006	0	0	0	0	0	0	1	0	0	0	0	0	1034
NM_182666	UBE2E1	Coding	-0.276	-0.394	150	-15.06	0	0.006	0	0	0	0	0	0	1	0	0	0	0	0	1034
NM_0012024	UBE2E1	Coding	-0.276	-0.394	150	-15.06	0	0.006	0	0	0	0	0	0	1	0	0	0	0	0	1034
NM_153183	NUDT10	Coding	-0.275	-0.343	153	-13.32	0	0.11	0	0	0	0	0	0	0	1	0	0	0	0	1286
NM_0013049	NUDT10	Coding	-0.275	-0.343	153	-13.32	0	0.11	0	0	0	0	0	0	0	1	0	0	0	0	1286
NM_021795	ELK4	Coding	-0.275	-0.366	148	-16	0	0.006	0	0	0	0	0	0	1	0	0	0	0	0	560
NM_032279	ATP13A4	Coding	-0.275	-0.339	154	-17.35	0	0.006	0	0	0	0	0	0	1	0	0	0	0	0	3685
NM_0012066	PSG7	Coding	-0.275	-0.325	146	-18.68	0	0.006	0	0	0	0	0	0	1	0	0	0	0	0	676
NM_0012900	PSG7	Coding	-0.275	-0.325	146	-18.68	0	0.006	0	0	0	0	0	0	1	0	0	0	0	0	676
NM_002783	PSG7	Coding	-0.275	-0.325	146	-18.68	0	0.006	0	0	0	0	0	0	1	0	0	0	0	0	676
NM_0012783	PHYKPL	Coding	-0.274	-0.346	140	-13.56	0	0.006	0	0	0	0	0	0	1	0	0	0	0	0	473
NM_153373	PHYKPL	Coding	-0.274	-0.346	140	-13.56	0	0.006	0	0	0	0	0	0	1	0	0	0	0	0	473
NM_0013010	VSTM2A	Coding	-0.274	-0.376	140	-13.9	0	0.006	0	0	0	0	0	0	1	0	0	0	0	0	1861
NM_0011950	ANKRD28	Coding	-0.273	-0.359	140	-12.51	0	0.006	0	0	0	0	0	0	1	0	0	0	0	0	3033
NM_0011950	ANKRD28	Coding	-0.273	-0.359	140	-12.51	0	0.006	0	0	0	0	0	0	1	0	0	0	0	0	3033
NM_015199	ANKRD28	Coding	-0.273	-0.359	140	-12.51	0	0.006	0	0	0	0	0	0	1	0	0	0	0	0	3033
NM_018696	ELAC1	Coding	-0.273	-0.354	146	-12.73	0	0.006	0	0	0	0	0	0	1	0	0	0	0	0	1035
NM_033379	CDK1	Coding	-0.273	-0.397	140	-17	0	0.006	0	0	0	0	0	0	1	0	0	0	0	0	877
NM_001786	CDK1	Coding	-0.273	-0.397	140	-17	0	0.006	0	0	0	0	0	0	1	0	0	0	0	0	877
NM_0013209	CDK1	Coding	-0.273	-0.397	140	-17	0	0.006	0	0	0	0	0	0	1	0	0	0	0	0	878
NM_019860	HTR7	Coding	-0.273	-0.324	144	-15.26	0	0.006	0	0	0	0	0	0	1	0	0	0	0	0	1711
NM_0013111	TNFAIP8L3	Coding	-0.273	-0.311	157	-21.08	0	0.006	0	0	0	0	0	0	1	0	0	0	0	0	1279
NM_207381	TNFAIP8L3	Coding	-0.273	-0.311	157	-21.08	0	0.006	0	0	0	0	0	0	1	0	0	0	0	0	1279
NM_015509	NECAP1	Coding	-0.272	-0.413	140	-11.75	0	0.006	0	0	0	0	0	0	1	0	0	0	0	0	1687
NM_001482	GATM	Coding	-0.272	-0.394	145	-11.39	0	0.006	0	0	0	0	0	0	1	0	0	0	0	0	985
NM_0013210	GATM	Coding	-0.272	-0.394	145	-11.39	0	0.006	0	0	0	0	0	0	1	0	0	0	0	0	985
NM_018218	USP40	Coding	-0.271	-0.349	153	-20.28	0	0.006	0	0	0	0	0	0	1	0	0	0	0	0	1873
NM_021083	XK	Coding	-0.271	-0.405	152	-15.97	0	0.006	0	0	0	0	0	0	1	0	0	0	0	0	3668
NM_0013082	IL2RA	Coding	-0.271	-0.397	145	-16.75	0	0.006	0	0	0	0	0	0	1	0	0	0	0	0	2178
NM_000417	IL2RA	Coding	-0.271	-0.397	145	-16.75	0	0.006	0	0	0	0	0	0	1	0	0	0	0	0	2178
NM_0013082	IL2RA	Coding	-0.271	-0.397	145	-16.75	0	0.006	0	0	0	0	0	0	1	0	0	0	0	0	2178
NM_032511	FAXC	Coding	-0.271	-0.386	153	-12.74	0	0.006	0	0	0	0	0	0	1	0	0	0	0	0	8247
NM_018440	PAG1	Coding	-0.269	-0.292	165	-25.97	0	0.006	0	0	0	0	0	0	1	0	0	0	0	0	8733
NM_0013190	EIF2A	Coding	-0.268	-0.296	143	-14.01	0	0.006	0	0	0	0	0	0	1	0	0	0	0	0	2105
NM_0013190	EIF2A	Coding	-0.268	-0.296	143	-14.01	0	0.006	0	0	0	0	0	0	1	0	0	0	0	0	2105
NM_0013190	EIF2A	Coding	-0.268	-0.296	143	-14.01	0	0.006	0	0	0	0	0	0	1	0	0	0	0	0	2105
NM_032025	EIF2A	Coding	-0.268	-0.296	143	-14.01	0	0.006	0	0	0	0	0	0	1	0	0	0	0	0	2105
NM_0013190	EIF2A	Coding	-0.268	-0.296	143	-14.01	0	0.006	0	0	0	0	0	0	1	0	0	0	0	0	2105
NM_0012571	LOC339862	Coding	-0.268	-0.403	153	-17.17	0	0.006	0	0	0	0	0	0	1	0	0	0	0	0	1875
NM_005240	ETV3	Coding	-0.268	-0.353	140	-14.08	0	0.006	0	0	0	0	0	0	1	0	0	0	0	0	896
NM_0013008	BATF2	Coding	-0.267	-0.373	154	-17.31	0	0.006	0	0	0	0	0	0	1	0	0	0	0	0	1184
NM_0013008	BATF2	Coding	-0.267	-0.373	154	-17.31	0	0.006	0	0	0	0	0	0	1	0	0	0	0	0	1184
NM_138456	BATF2	Coding	-0.267	-0.373	154	-17.31	0	0.006	0	0	0	0	0	0	1	0	0	0	0	0	1184
NM_0011024	TTI2	Coding	-0.267	-0.299	156	-15.28	0	0.006	0	0	0	0	0	0	1	0	0	0	0	0	664
NM_0012655	TTI2	Coding	-0.267	-0.299	156	-15.28	0	0.006	0	0	0	0	0	0	1	0	0	0	0	0	664
NM_025115	TTI2	Coding	-0.267	-0.299	156	-15.28	0	0.006	0	0	0	0	0	0	1	0	0	0	0	0	664
NM_005584	MAB21L1	Coding	-0.267	-0.357	142	-11.88	0	0.006	0	0	0	0	0	0	1	0	0	0	0	0	1270
NM_014062	NOB1	Coding	-0.267	-0.293	145	-18.11	0	0.006	0	0	0	0	0	0	1	0	0	0	0	0	478

NM_003226	TFF3	Coding	-0.266	-0.299	154	-16.12	0	0.006	0	0	0	0	0	0	1	0	0	0	0	0	589
NM_032693	NAA11	Coding	-0.266	-0.288	153	-19.21	0	0.006	0	0	0	0	0	0	1	0	0	0	0	0	686
NM_001130(	ELMOD1	Coding	-0.266	-0.363	174	-24.01	0	0.11	0	0	0	0	0	0	0	1	0	0	0	0	1582
NM_001308(	ELMOD1	Coding	-0.266	-0.363	174	-24.01	0	0.11	0	0	0	0	0	0	0	1	0	0	0	0	1582
NM_018712	ELMOD1	Coding	-0.266	-0.363	174	-24.01	0	0.11	0	0	0	0	0	0	0	1	0	0	0	0	1582
NM_001135(	ARHGAP26	Coding	-0.265	-0.317	146	-15.06	0	0.006	0	0	0	0	0	0	1	0	0	0	0	0	6561
NM_015071	ARHGAP26	Coding	-0.265	-0.317	146	-15.06	0	0.006	0	0	0	0	0	0	1	0	0	0	0	0	6561
NM_014149	WDR91	Coding	-0.265	-0.357	140	-12.03	0	0.006	0	0	0	0	0	0	1	0	0	0	0	0	2313
NM_015933	TMA7	Coding	-0.264	-0.348	164	-21.1	0	0.11	0	0	0	0	0	0	0	1	0	0	0	0	247
NM_001018(	SFMBT2	Coding	-0.264	-0.401	140	-13.02	0	0.006	0	0	0	0	0	0	1	0	0	0	0	0	5146
NM_001029(	SFMBT2	Coding	-0.264	-0.401	140	-13.02	0	0.006	0	0	0	0	0	0	1	0	0	0	0	0	5146
NM_001128(	DIS3	Coding	-0.264	-0.301	159	-22.24	0	0.006	0	0	0	0	0	0	1	0	0	0	0	0	4393
NM_014953	DIS3	Coding	-0.264	-0.301	159	-22.24	0	0.006	0	0	0	0	0	0	1	0	0	0	0	0	4393
NM_001322(	DIS3	Coding	-0.264	-0.301	159	-22.24	0	0.006	0	0	0	0	0	0	1	0	0	0	0	0	4393
NM_001322(	DIS3	Coding	-0.264	-0.301	159	-22.24	0	0.006	0	0	0	0	0	0	1	0	0	0	0	0	4393
NM_138447	ZNF689	Coding	-0.263	-0.328	149	-16.49	0	0.006	0	0	0	0	0	0	1	0	0	0	0	0	1706
NM_198992	SYT10	Coding	-0.262	-0.415	155	-18.17	0	0.006	0	0	0	0	0	0	1	0	0	0	0	0	1417
NM_173631	ZNF547	Coding	-0.262	-0.349	142	-12.89	0	0.006	0	0	0	0	0	0	1	0	0	0	0	0	1372
NM_005986	SOX1	Coding	-0.262	-0.334	153	-19.12	0	0.006	0	0	0	0	0	0	1	0	0	0	0	0	2872
NM_024949	WWC2	Coding	-0.261	-0.335	157	-17.14	0	0.11	0	0	0	0	0	0	0	1	0	0	0	0	5047
NM_020697	KCNS2	Coding	-0.261	-0.373	156	-17.41	0	0.006	0	0	0	0	0	0	1	0	0	0	0	0	1382
NM_001206(	RIC1	Coding	-0.259	-0.352	161	-19.74	0	0.006	0	0	0	0	0	0	1	0	0	0	0	0	2310
NM_020829	RIC1	Coding	-0.259	-0.352	161	-19.74	0	0.006	0	0	0	0	0	0	1	0	0	0	0	0	2310
NM_001730	KLF5	Coding	-0.258	-0.293	146	-15.5	0	0.006	0	0	0	0	0	0	1	0	0	0	0	0	1656
NM_001286(	KLF5	Coding	-0.258	-0.293	146	-15.5	0	0.006	0	0	0	0	0	0	1	0	0	0	0	0	1656
NM_145872	ASB4	Coding	-0.257	-0.288	148	-16.03	0	0.006	0	0	0	0	0	0	1	0	0	0	0	0	426
NM_002079	GOT1	Coding	-0.256	-0.335	148	-11.75	0	0.006	0	0	0	0	0	0	1	0	0	0	0	0	677
NM_001278(	SLC38A1	Coding	-0.255	-0.281	157	-22.24	0	0.006	0	0	0	0	0	0	1	0	0	0	0	0	402
NM_001282(	FAM71F1	Coding	-0.254	-0.263	145	-15.48	0	0.006	0	0	0	0	0	0	1	0	0	0	0	0	630
NM_001282(	FAM71F1	Coding	-0.254	-0.263	145	-15.48	0	0.006	0	0	0	0	0	0	1	0	0	0	0	0	630
NM_032599	FAM71F1	Coding	-0.254	-0.263	145	-15.48	0	0.006	0	0	0	0	0	0	1	0	0	0	0	0	630
NM_001015(	PHF6	Coding	-0.254	-0.392	146	-14.44	0	0.006	0	0	0	0	0	0	1	0	0	0	0	0	3132
NM_032458	PHF6	Coding	-0.254	-0.392	146	-14.44	0	0.006	0	0	0	0	0	0	1	0	0	0	0	0	3462
NM_001319(	CLEC12B	Coding	-0.254	-0.338	162	-22.21	0	0.11	0	0	0	0	0	0	0	1	0	0	0	0	2203
NM_205852	CLEC12B	Coding	-0.254	-0.338	162	-22.21	0	0.11	0	0	0	0	0	0	0	1	0	0	0	0	2203
NM_032656	DHX37	Coding	-0.254	-0.344	151	-16.02	0	0.006	0	0	0	0	0	0	1	0	0	0	0	0	978
NM_002076	GNS	Coding	-0.254	-0.297	154	-17.5	0	0.006	0	0	0	0	0	0	1	0	0	0	0	0	3299
NM_002486	NCBP1	Coding	-0.253	-0.389	144	-11.79	0	0.006	0	0	0	0	0	0	1	0	0	0	0	0	2548
NM_178128	FADS6	Coding	-0.252	-0.289	151	-20.08	0	0.006	0	0	0	0	0	0	1	0	0	0	0	0	1018
NM_006273	CCL7	Coding	-0.25	-0.315	145	-13.28	0	0.006	0	0	0	0	0	0	1	0	0	0	0	0	440
NM_018260	ZNF701	Coding	-0.25	-0.359	140	-11.75	0	0.006	0	0	0	0	0	0	1	0	0	0	0	0	3717
NM_001172(	ZNF701	Coding	-0.25	-0.359	140	-11.75	0	0.006	0	0	0	0	0	0	1	0	0	0	0	0	3717
NM_017420	SIX4	Coding	-0.25	-0.416	140	-15.27	0	0.006	0	0	0	0	0	0	1	0	0	0	0	0	3869
NM_002355	M6PR	Coding	-0.25	-0.233	166	-19.66	0	0.006	0	0	0	0	0	0	1	0	0	0	0	0	1457
NM_001207(	M6PR	Coding	-0.25	-0.233	166	-19.66	0	0.006	0	0	0	0	0	0	1	0	0	0	0	0	1457
NM_001111(	CLDN22	Coding	-0.25	-0.301	142	-15.1	0	0.006	0	0	0	0	0	0	1	0	0	0	0	0	1489
NM_015986	CRLF3	Coding	-0.249	-0.311	146	-14.24	0	0.11	0	0	0	0	0	0	0	1	0	0	0	0	1503
NM_004210	NEURL1	Coding	-0.249	-0.318	142	-16.41	0	0.006	0	0	0	0	0	0	1	0	0	0	0	0	2180
NM_000928	PLA2G1B	Coding	-0.249	-0.263	156	-18.24	0	0.006	0	0	0	0	0	0	1	0	0	0	0	0	82
NM_017882	CLN6	Coding	-0.249	-0.335	144	-12.75	0	0.006	0	0	0	0	0	0	1	0	0	0	0	0	1148
NM_001321(	RAD51B	Coding	-0.249	-0.277	150	-15.08	0	0.006	0	0	0	0	0	0	1	0	0	0	0	0	1879
NM_000771	CYP2C9	Coding	-0.248	-0.305	141	-13.11	0	0.006	0	0	0	0	0	0	1	0	0	0	0	0	363
NM_001143(	RBM24	Coding	-0.248	-0.405	147	-14.92	0	0.006	0	0	0	0	0	0	1	0	0	0	0	0	1749
NM_001143(	RBM24	Coding	-0.248	-0.405	147	-14.92	0	0.006	0	0	0	0	0	0	1	0	0	0	0	0	1749
NM_153020	RBM24	Coding	-0.248	-0.405	147	-14.92	0	0.006	0	0	0	0	0	0	1	0	0	0	0	0	1749
NM_022748	TNS3	Coding	-0.248	-0.292	140	-10.58	0	0.11	0	0	0	0	0	0	0	1	0	0	0	0	2922
NM_001199(	NCOA7	Coding	-0.248	-0.31	143	-13.8	0	0.006	0	0	0	0	0	0	1	0	0	0	0	0	3259
NM_001199(	NCOA7	Coding	-0.248	-0.31	143	-13.8	0	0.006	0	0	0	0	0	0	1	0	0	0	0	0	3259
NM_001122(	NCOA7	Coding	-0.248	-0.31	143	-13.8	0	0.006	0	0	0	0	0	0	1	0	0	0	0	0	3259
NM_001199(	NCOA7	Coding	-0.248	-0.31	143	-13.8	0	0.006	0	0	0	0	0	0	1	0	0	0	0	0	3259

NM_181782	NCOA7	Coding	-0.248	-0.31	143	-13.8	0	0.006	0	0	0	0	0	0	1	0	0	0	0	0	3259
NM_0011996	NCOA7	Coding	-0.248	-0.31	143	-13.8	0	0.006	0	0	0	0	0	0	1	0	0	0	0	0	3259
NM_145332	MAP3K7	Coding	-0.248	-0.387	146	-12.44	0	0.006	0	0	0	0	0	0	1	0	0	0	0	0	3076
NM_145333	MAP3K7	Coding	-0.248	-0.387	146	-12.44	0	0.006	0	0	0	0	0	0	1	0	0	0	0	0	3076
NM_006626	ZBTB6	Coding	-0.248	-0.325	148	-16.09	0	0.006	0	0	0	0	0	0	1	0	0	0	0	0	2742
NM_0012014	NETO2	Coding	-0.247	-0.326	142	-10.45	0	0.11	0	0	0	0	0	0	0	1	0	0	0	0	1701
NM_018092	NETO2	Coding	-0.247	-0.326	142	-10.45	0	0.11	0	0	0	0	0	0	0	1	0	0	0	0	1701
NM_203423	LOC389199	Coding	-0.247	-0.213	152	-22.93	0	0.006	0	0	0	0	0	0	1	0	0	0	0	0	315
NM_198057	TSC22D3	Coding	-0.246	-0.298	147	-15.77	0	0.006	0	0	0	0	0	0	1	0	0	0	0	0	1297
NM_0013184	TSC22D3	Coding	-0.246	-0.298	147	-15.77	0	0.006	0	0	0	0	0	0	1	0	0	0	0	0	1297
NM_0013184	TSC22D3	Coding	-0.246	-0.298	147	-15.77	0	0.006	0	0	0	0	0	0	1	0	0	0	0	0	1297
NM_004089	TSC22D3	Coding	-0.246	-0.298	147	-15.77	0	0.006	0	0	0	0	0	0	1	0	0	0	0	0	1297
NM_0010158	TSC22D3	Coding	-0.246	-0.298	147	-15.77	0	0.006	0	0	0	0	0	0	1	0	0	0	0	0	1297
NM_0010853	BTLA	Coding	-0.246	-0.321	161	-16.3	0	0.11	0	0	0	0	0	0	0	1	0	0	0	0	2142
NM_181780	BTLA	Coding	-0.246	-0.321	161	-16.3	0	0.11	0	0	0	0	0	0	0	1	0	0	0	0	2142
NM_0012786	NFXL1	Coding	-0.246	-0.312	147	-12.61	0	0.11	0	0	0	0	0	0	0	1	0	0	0	0	930
NM_152995	NFXL1	Coding	-0.246	-0.312	147	-12.61	0	0.11	0	0	0	0	0	0	0	1	0	0	0	0	930
NM_0012786	NFXL1	Coding	-0.246	-0.312	147	-12.61	0	0.11	0	0	0	0	0	0	0	1	0	0	0	0	930
NM_004857	AKAP5	Coding	-0.246	-0.307	143	-11.51	0	0.11	0	0	0	0	0	0	0	1	0	0	0	0	4825
NM_0011451	TCP10L2	Coding	-0.246	-0.253	165	-20.86	0	0.006	0	0	0	0	0	0	1	0	0	0	0	0	992
NM_080721	OCSTAMP	Coding	-0.245	-0.239	150	-21.34	0	0.006	0	0	0	0	0	0	1	0	0	0	0	0	243
NM_0010322	STXBP1	Coding	-0.245	-0.313	146	-9.96	0	0.11	0	0	0	0	0	0	0	1	0	0	0	0	1859
NM_003165	STXBP1	Coding	-0.245	-0.313	146	-9.96	0	0.11	0	0	0	0	0	0	0	1	0	0	0	0	1958
NM_0012426	ENO4	Coding	-0.245	-0.37	146	-18.09	0	0.006	0	0	0	0	0	0	1	0	0	0	0	0	932
NM_005420	SULT1E1	Coding	-0.245	-0.303	143	-12.02	0	0.11	0	0	0	0	0	0	0	1	0	0	0	0	782
NM_004798	KIF3B	Coding	-0.244	-0.295	148	-11.75	0	0.11	0	0	0	0	0	0	0	1	0	0	0	0	3689
NM_018683	RNF114	Coding	-0.244	-0.336	140	-14.44	0	0.006	0	0	0	0	0	0	1	0	0	0	0	0	1744
NM_0012718	RGS7BP	Coding	-0.244	-0.278	163	-22.85	0	0.006	0	0	0	0	0	0	1	0	0	0	0	0	3130
NM_0011994	BRD2	Coding	-0.244	-0.24	167	-17.5	0	0.006	0	0	0	0	0	0	1	0	0	0	0	0	787
NM_0011994	BRD2	Coding	-0.244	-0.24	167	-17.5	0	0.006	0	0	0	0	0	0	1	0	0	0	0	0	787
NM_005104	BRD2	Coding	-0.244	-0.24	167	-17.5	0	0.006	0	0	0	0	0	0	1	0	0	0	0	0	787
NM_0012919	BRD2	Coding	-0.244	-0.24	167	-17.5	0	0.006	0	0	0	0	0	0	1	0	0	0	0	0	787
NM_0011131	BRD2	Coding	-0.244	-0.24	167	-17.5	0	0.006	0	0	0	0	0	0	1	0	0	0	0	0	787
NM_004738	VAPB	Coding	-0.243	-0.419	158	-15.67	0	0.006	0	0	0	0	0	0	1	0	0	0	0	0	6865
NM_0012878	ZWILCH	Coding	-0.243	-0.398	145	-14.47	0	0.006	0	0	0	0	0	0	1	0	0	0	0	0	1160
NM_0012878	ZWILCH	Coding	-0.243	-0.398	145	-14.47	0	0.006	0	0	0	0	0	0	1	0	0	0	0	0	1160
NM_0012878	ZWILCH	Coding	-0.243	-0.398	145	-14.47	0	0.006	0	0	0	0	0	0	1	0	0	0	0	0	1160
NM_017975	ZWILCH	Coding	-0.243	-0.398	145	-14.47	0	0.006	0	0	0	0	0	0	1	0	0	0	0	0	1160
NM_0013008	C1orf162	Coding	-0.243	-0.282	143	-11.9	0	0.006	0	0	0	0	0	0	1	0	0	0	0	0	389
NM_0013008	C1orf162	Coding	-0.243	-0.282	143	-11.9	0	0.006	0	0	0	0	0	0	1	0	0	0	0	0	389
NM_174896	C1orf162	Coding	-0.243	-0.282	143	-11.9	0	0.006	0	0	0	0	0	0	1	0	0	0	0	0	389
NM_014616	ATP11B	Coding	-0.241	-0.321	154	-15.94	0	0.11	0	0	0	0	0	0	0	1	0	0	0	0	3529
NM_0010997	PSMB11	Coding	-0.24	-0.329	151	-13.34	0	0.006	0	0	0	0	0	0	1	0	0	0	0	0	932
NM_182546	VSTM2A	Coding	-0.24	-0.353	140	-13.9	0	0.006	0	0	0	0	0	0	1	0	0	0	0	0	1995
NM_004882	CIR1	Coding	-0.24	-0.295	154	-11.16	0	0.11	0	0	0	0	0	0	0	1	0	0	0	0	347
NM_020873	LRRN1	Coding	-0.239	-0.287	147	-12.76	0	0.11	0	0	0	0	0	0	0	1	0	0	0	0	3042
NM_177995	PTPDC1	Coding	-0.238	-0.303	147	-14.67	0	0.006	0	0	0	0	0	0	1	0	0	0	0	0	1912
NM_0012538	PTPDC1	Coding	-0.238	-0.303	147	-14.67	0	0.006	0	0	0	0	0	0	1	0	0	0	0	0	1912
NM_0012538	PTPDC1	Coding	-0.238	-0.303	147	-14.67	0	0.006	0	0	0	0	0	0	1	0	0	0	0	0	1912
NM_152422	PTPDC1	Coding	-0.238	-0.303	147	-14.67	0	0.006	0	0	0	0	0	0	1	0	0	0	0	0	1912
NM_017507	ZNF823	Coding	-0.238	-0.309	152	-16.71	0	0.11	0	0	0	0	0	0	0	1	0	0	0	0	436
NM_0010804	ZNF823	Coding	-0.238	-0.309	152	-16.71	0	0.11	0	0	0	0	0	0	0	1	0	0	0	0	436
NM_0012976	ZNF823	Coding	-0.238	-0.309	152	-16.71	0	0.11	0	0	0	0	0	0	0	1	0	0	0	0	436
NM_0013219	ZFP82	Coding	-0.238	-0.354	158	-19.6	0	0.006	0	0	0	0	0	0	1	0	0	0	0	0	4111
NM_133466	ZFP82	Coding	-0.238	-0.354	158	-19.6	0	0.006	0	0	0	0	0	0	1	0	0	0	0	0	4111
NM_001537	HSBP1	Coding	-0.237	-0.316	152	-13.15	0	0.006	0	0	0	0	0	0	1	0	0	0	0	0	1584
NM_177925	H2AFJ	Coding	-0.237	-0.326	153	-15.57	0	0.11	0	0	0	0	0	0	0	1	0	0	0	0	3142
NM_000415	IAPP	Coding	-0.237	-0.315	159	-20.41	0	0.11	0	0	0	0	0	0	0	1	0	0	0	0	1554
NM_178129	P2RY8	Coding	-0.236	-0.313	147	-18.55	0	0.11	0	0	0	0	0	0	0	1	0	0	0	0	2906
NM_005044	PRKX	Coding	-0.236	-0.4	140	-11.75	0	0.006	0	0	0	0	0	0	1	0	0	0	0	0	4626

NM_021238	FAM60A	Coding	-0.236	-0.313	149	-14.57	0	0.11	0	0	0	0	0	0	0	1	0	0	0	0	2126
NM_0011358	FAM60A	Coding	-0.236	-0.313	149	-14.57	0	0.11	0	0	0	0	0	0	0	1	0	0	0	0	2126
NM_0011358	FAM60A	Coding	-0.236	-0.313	149	-14.57	0	0.11	0	0	0	0	0	0	0	1	0	0	0	0	2126
NM_0013080	STARD4	Coding	-0.236	-0.293	145	-10.98	0	0.11	0	0	0	0	0	0	0	1	0	0	0	0	3852
NM_0013080	STARD4	Coding	-0.236	-0.293	145	-10.98	0	0.11	0	0	0	0	0	0	0	1	0	0	0	0	3852
NM_0013080	STARD4	Coding	-0.236	-0.293	145	-10.98	0	0.11	0	0	0	0	0	0	0	1	0	0	0	0	3852
NM_0013080	STARD4	Coding	-0.236	-0.293	145	-10.98	0	0.11	0	0	0	0	0	0	0	1	0	0	0	0	3852
NM_139164	STARD4	Coding	-0.236	-0.293	145	-10.98	0	0.11	0	0	0	0	0	0	0	1	0	0	0	0	3852
NM_0011647	ASPH	Coding	-0.235	-0.28	166	-22.82	0	0.006	0	0	0	0	0	0	1	0	0	0	0	0	2803
NM_004318	ASPH	Coding	-0.235	-0.28	166	-22.82	0	0.006	0	0	0	0	0	0	1	0	0	0	0	0	2803
NM_000893	KNG1	Coding	-0.234	-0.283	140	-9.57	0	0.11	0	0	0	0	0	0	0	1	0	0	0	0	625
NM_0011664	KNG1	Coding	-0.234	-0.283	140	-9.57	0	0.11	0	0	0	0	0	0	0	1	0	0	0	0	625
NM_014639	TTC37	Coding	-0.234	-0.31	165	-16.73	0	0.11	0	0	0	0	0	0	0	1	0	0	0	0	712
NM_0011423	DMTF1	Coding	-0.234	-0.288	145	-12.7	0	0.006	0	0	0	0	0	0	1	0	0	0	0	0	1192
NM_0011423	DMTF1	Coding	-0.234	-0.288	145	-12.7	0	0.006	0	0	0	0	0	0	1	0	0	0	0	0	1192
NM_021145	DMTF1	Coding	-0.234	-0.288	145	-12.7	0	0.006	0	0	0	0	0	0	1	0	0	0	0	0	1192
NM_182832	PLAC4	Coding	-0.234	-0.316	140	-12.34	0	0.006	0	0	0	0	0	0	1	0	0	0	0	0	3945
NM_016094	COMMD2	Coding	-0.233	-0.286	146	-10.52	0	0.11	0	0	0	0	0	0	0	1	0	0	0	0	3051
NM_079834	SCAMP4	Coding	-0.233	-0.268	153	-16.81	0	0.006	0	0	0	0	0	0	1	0	0	0	0	0	1729
NM_181727	SPATA12	Coding	-0.232	-0.321	158	-15.86	0	0.11	0	0	0	0	0	0	0	1	0	0	0	0	1165
NM_173799	TIGIT	Coding	-0.232	-0.286	152	-21.57	0	0.006	0	0	0	0	0	0	1	0	0	0	0	0	2157
NM_0013163	ITGA6	Coding	-0.232	-0.343	143	-13.67	0	0.006	0	0	0	0	0	0	1	0	0	0	0	0	2387
NM_000210	ITGA6	Coding	-0.232	-0.343	143	-13.67	0	0.006	0	0	0	0	0	0	1	0	0	0	0	0	2387
NM_0010798	ITGA6	Coding	-0.232	-0.343	143	-13.67	0	0.006	0	0	0	0	0	0	1	0	0	0	0	0	2203
NM_152280	SYT11	Coding	-0.231	-0.303	146	-16.55	0	0.006	0	0	0	0	0	0	1	0	0	0	0	0	3691
NM_0011438	HRH4	Coding	-0.229	-0.313	152	-16.86	0	0.11	0	0	0	0	0	0	0	1	0	0	0	0	2395
NM_021624	HRH4	Coding	-0.229	-0.313	152	-16.86	0	0.11	0	0	0	0	0	0	0	1	0	0	0	0	2395
NM_004979	KCND1	Coding	-0.229	-0.273	150	-18.91	0	0.006	0	0	0	0	0	0	1	0	0	0	0	0	1203
NM_024331	TTPAL	Coding	-0.229	-0.323	150	-20.14	0	0.006	0	0	0	0	0	0	1	0	0	0	0	0	5062
NM_0012618	TTPAL	Coding	-0.229	-0.323	150	-20.14	0	0.006	0	0	0	0	0	0	1	0	0	0	0	0	5062
NM_0010391	TTPAL	Coding	-0.229	-0.323	150	-20.14	0	0.006	0	0	0	0	0	0	1	0	0	0	0	0	5062
NM_005898	CAPRIN1	Coding	-0.228	-0.415	140	-11.75	0	0.006	0	0	0	0	0	0	1	0	0	0	0	0	3243
NM_0013032	MORC2	Coding	-0.228	-0.271	151	-17.4	0	0.006	0	0	0	0	0	0	1	0	0	0	0	0	1673
NM_0013032	MORC2	Coding	-0.228	-0.271	151	-17.4	0	0.006	0	0	0	0	0	0	1	0	0	0	0	0	1673
NM_014941	MORC2	Coding	-0.228	-0.271	151	-17.4	0	0.006	0	0	0	0	0	0	1	0	0	0	0	0	1673
NM_0011350	MAPK9	Coding	-0.227	-0.277	154	-15.89	0	0.006	0	0	0	0	0	0	1	0	0	0	0	0	1370
NM_000868	HTR2C	Coding	-0.226	-0.293	149	-15.13	0	0.006	0	0	0	0	0	0	1	0	0	0	0	0	2646
NM_0012567	HTR2C	Coding	-0.226	-0.293	149	-15.13	0	0.006	0	0	0	0	0	0	1	0	0	0	0	0	2646
NM_004854	CHST10	Coding	-0.226	-0.331	146	-13.29	0	0.006	0	0	0	0	0	0	1	0	0	0	0	0	1385
NM_052848	CCDC97	Coding	-0.226	-0.238	147	-17.51	0	0.006	0	0	0	0	0	0	1	0	0	0	0	0	2168
NM_001755	CBFB	Coding	-0.226	-0.275	145	-11.58	0	0.11	0	0	0	0	0	0	0	1	0	0	0	0	2323
NM_022845	CBFB	Coding	-0.226	-0.275	145	-11.58	0	0.11	0	0	0	0	0	0	0	1	0	0	0	0	2277
NM_0012862	GOT2	Coding	-0.226	-0.215	153	-17.86	0	0.006	0	0	0	0	0	0	1	0	0	0	0	0	1040
NM_002080	GOT2	Coding	-0.226	-0.215	153	-17.86	0	0.006	0	0	0	0	0	0	1	0	0	0	0	0	1040
NM_001277	CHKA	Coding	-0.226	-0.289	154	-18.93	0	0.11	0	0	0	0	0	0	0	1	0	0	0	0	1129
NM_212469	CHKA	Coding	-0.226	-0.289	154	-18.93	0	0.11	0	0	0	0	0	0	0	1	0	0	0	0	1129
NM_0013187	TLR2	Coding	-0.225	-0.311	145	-10.47	0	0.11	0	0	0	0	0	0	0	1	0	0	0	0	998
NM_0013187	TLR2	Coding	-0.225	-0.311	145	-10.47	0	0.11	0	0	0	0	0	0	0	1	0	0	0	0	998
NM_0013187	TLR2	Coding	-0.225	-0.311	145	-10.47	0	0.11	0	0	0	0	0	0	0	1	0	0	0	0	998
NM_0013187	TLR2	Coding	-0.225	-0.311	145	-10.47	0	0.11	0	0	0	0	0	0	0	1	0	0	0	0	998
NM_0013187	TLR2	Coding	-0.225	-0.311	145	-10.47	0	0.11	0	0	0	0	0	0	0	1	0	0	0	0	998
NM_0013187	TLR2	Coding	-0.225	-0.311	145	-10.47	0	0.11	0	0	0	0	0	0	0	1	0	0	0	0	998
NM_0013187	TLR2	Coding	-0.225	-0.311	145	-10.47	0	0.11	0	0	0	0	0	0	0	1	0	0	0	0	998
NM_003264	TLR2	Coding	-0.225	-0.311	145	-10.47	0	0.11	0	0	0	0	0	0	0	1	0	0	0	0	998
NM_183044	RNF6	Coding	-0.225	-0.338	145	-13.97	0	0.006	0	0	0	0	0	0	1	0	0	0	0	0	1056
NM_005977	RNF6	Coding	-0.225	-0.338	145	-13.97	0	0.006	0	0	0	0	0	0	1	0	0	0	0	0	1056
NM_183043	RNF6	Coding	-0.225	-0.338	145	-13.97	0	0.006	0	0	0	0	0	0	1	0	0	0	0	0	1056
NM_000903	NQO1	Coding	-0.225	-0.321	142	-15.63	0	0.006	0	0	0	0	0	0	1	0	0	0	0	0	1575
NM_0010254	NQO1	Coding	-0.225	-0.321	142	-15.63	0	0.006	0	0	0	0	0	0	1	0	0	0	0	0	1575
NM_0010254	NQO1	Coding	-0.225	-0.321	142	-15.63	0	0.006	0	0	0	0	0	0	1	0	0	0	0	0	1575

NM_0012861	NQO1	Coding	-0.225	-0.321	142	-15.63	0	0.006	0	0	0	0	0	0	1	0	0	0	0	0	1575
NM_001195(	VAPB	Coding	-0.225	-0.407	158	-15.67	0	0.006	0	0	0	0	0	0	1	0	0	0	0	0	6935
NM_144975	SLFN5	Coding	-0.225	-0.285	151	-13.92	0	0.11	0	0	0	0	0	0	0	1	0	0	0	0	1854
NM_0013082	MAPK9	Coding	-0.225	-0.275	154	-15.89	0	0.006	0	0	0	0	0	0	1	0	0	0	0	0	1528
NM_019601	SUSD2	Coding	-0.225	-0.22	151	-16.48	0	0.006	0	0	0	0	0	0	1	0	0	0	0	0	670
NM_024901	DENND2D	Coding	-0.225	-0.303	145	-11.69	0	0.11	0	0	0	0	0	0	0	1	0	0	0	0	1636
NM_0012718	DENND2D	Coding	-0.225	-0.303	145	-11.69	0	0.11	0	0	0	0	0	0	0	1	0	0	0	0	1636
NM_020848	KIAA1462	Coding	-0.224	-0.318	149	-16.33	0	0.11	0	0	0	0	0	0	0	1	0	0	0	0	5083
NM_0011233	CCR2	Coding	-0.224	-0.238	147	-18.21	0	0.006	0	0	0	0	0	0	1	0	0	0	0	0	767
NM_001308(	TICRR	Coding	-0.224	-0.329	150	-15.07	0	0.11	0	0	0	0	0	0	0	1	0	0	0	0	936
NM_152259	TICRR	Coding	-0.224	-0.329	150	-15.07	0	0.11	0	0	0	0	0	0	0	1	0	0	0	0	936
NM_002119	HLA-DOA	Coding	-0.224	-0.339	146	-16.39	0	0.006	0	0	0	0	0	0	1	0	0	0	0	0	2655
NM_0010831	GPD2	Coding	-0.223	-0.304	166	-30.59	0	0.006	0	0	0	0	0	0	1	0	0	0	0	0	3485
NM_000408	GPD2	Coding	-0.223	-0.304	166	-30.59	0	0.006	0	0	0	0	0	0	1	0	0	0	0	0	3485
NM_031412	GABARAPL1	Coding	-0.223	-0.29	151	-11.6	0	0.11	0	0	0	0	0	0	0	1	0	0	0	0	1273
NM_177531	PKHD1L1	Coding	-0.223	-0.298	144	-13.39	0	0.006	0	0	0	0	0	0	1	0	0	0	0	0	1181
NM_001145(	PTPRQ	Coding	-0.223	-0.339	148	-18.62	0	0.006	0	0	0	0	0	0	1	0	0	0	0	0	1166
NM_0013222	DHX8	Coding	-0.223	-0.256	148	-18.62	0	0.006	0	0	0	0	0	0	1	0	0	0	0	0	588
NM_004783	TAOK2	Coding	-0.223	-0.215	147	-15.35	0	0.006	0	0	0	0	0	0	1	0	0	0	0	0	693
NM_001249	ENTPD5	Coding	-0.222	-0.374	146	-14.84	0	0.006	0	0	0	0	0	0	1	0	0	0	0	0	3697
NM_0013219	ENTPD5	Coding	-0.222	-0.374	146	-14.84	0	0.006	0	0	0	0	0	0	1	0	0	0	0	0	3697
NM_0013219	ENTPD5	Coding	-0.222	-0.374	146	-14.84	0	0.006	0	0	0	0	0	0	1	0	0	0	0	0	3697
NM_0013219	ENTPD5	Coding	-0.222	-0.374	146	-14.84	0	0.006	0	0	0	0	0	0	1	0	0	0	0	0	3697
NM_0013219	ENTPD5	Coding	-0.222	-0.374	146	-14.84	0	0.006	0	0	0	0	0	0	1	0	0	0	0	0	3697
NM_139131	NUP98	Coding	-0.222	-0.271	153	-13.19	0	0.11	0	0	0	0	0	0	0	1	0	0	0	0	989
NM_005387	NUP98	Coding	-0.222	-0.271	153	-13.19	0	0.11	0	0	0	0	0	0	0	1	0	0	0	0	989
NM_0012866	MLLT3	Coding	-0.221	-0.296	153	-16.3	0	0.11	0	0	0	0	0	0	0	1	0	0	0	0	4778
NM_004529	MLLT3	Coding	-0.221	-0.296	153	-16.3	0	0.11	0	0	0	0	0	0	0	1	0	0	0	0	4778
NM_0013035	VBP1	Coding	-0.221	-0.273	153	-10.3	0	0.11	0	0	0	0	0	0	0	1	0	0	0	0	999
NM_003372	VBP1	Coding	-0.221	-0.273	153	-10.3	0	0.11	0	0	0	0	0	0	0	1	0	0	0	0	999
NM_0013035	VBP1	Coding	-0.221	-0.273	153	-10.3	0	0.11	0	0	0	0	0	0	0	1	0	0	0	0	999
NM_0013035	VBP1	Coding	-0.221	-0.273	153	-10.3	0	0.11	0	0	0	0	0	0	0	1	0	0	0	0	999
NM_017414	USP18	Coding	-0.221	-0.265	143	-9.76	0	0.11	0	0	0	0	0	0	0	1	0	0	0	0	578
NM_0011676	CADM2	Coding	-0.22	-0.299	155	-13.56	0	0.11	0	0	0	0	0	0	0	1	0	0	0	0	7646
NM_0011676	CADM2	Coding	-0.22	-0.299	155	-13.56	0	0.11	0	0	0	0	0	0	0	1	0	0	0	0	7646
NM_0012565	CADM2	Coding	-0.22	-0.299	155	-13.56	0	0.11	0	0	0	0	0	0	0	1	0	0	0	0	7646
NM_0012565	CADM2	Coding	-0.22	-0.299	155	-13.56	0	0.11	0	0	0	0	0	0	0	1	0	0	0	0	7646
NM_0012565	CADM2	Coding	-0.22	-0.299	155	-13.56	0	0.11	0	0	0	0	0	0	0	1	0	0	0	0	7646
NM_0012565	CADM2	Coding	-0.22	-0.299	155	-13.56	0	0.11	0	0	0	0	0	0	0	1	0	0	0	0	7646
NM_153184	CADM2	Coding	-0.22	-0.299	155	-13.56	0	0.11	0	0	0	0	0	0	0	1	0	0	0	0	7646
NM_001318(	ZNF514	Coding	-0.22	-0.341	165	-23.52	0	0.006	0	0	0	0	0	0	1	0	0	0	0	0	4282
NM_032788	ZNF514	Coding	-0.22	-0.341	165	-23.52	0	0.006	0	0	0	0	0	0	1	0	0	0	0	0	4282
NM_000383	AIRE	Coding	-0.22	-0.267	143	-11.69	0	0.11	0	0	0	0	0	0	0	1	0	0	0	0	492
NM_024819	DCAKD	Coding	-0.22	-0.29	155	-21.05	0	0.11	0	0	0	0	0	0	0	1	0	0	0	0	1095
NM_0012886	DCAKD	Coding	-0.22	-0.29	155	-21.05	0	0.11	0	0	0	0	0	0	0	1	0	0	0	0	1095
NM_0012886	DCAKD	Coding	-0.22	-0.29	155	-21.05	0	0.11	0	0	0	0	0	0	0	1	0	0	0	0	1095
NM_0013213	DCAKD	Coding	-0.22	-0.29	155	-21.05	0	0.11	0	0	0	0	0	0	0	1	0	0	0	0	1095
NM_0011286	DCAKD	Coding	-0.22	-0.29	155	-21.05	0	0.11	0	0	0	0	0	0	0	1	0	0	0	0	1095
NM_133379	TTN	Coding	-0.22	-0.319	156	-14.42	0	0.11	0	0	0	0	0	0	0	1	0	0	0	0	1176
NM_0010098	C12orf29	Coding	-0.22	-0.308	154	-11.76	0	0.11	0	0	0	0	0	0	0	1	0	0	0	0	1738
NM_001030(	AP2B1	Coding	-0.22	-0.284	163	-21.86	0	0.006	0	0	0	0	0	0	1	0	0	0	0	0	2718
NM_001282	AP2B1	Coding	-0.22	-0.284	163	-21.86	0	0.006	0	0	0	0	0	0	1	0	0	0	0	0	2718
NM_0011451	HBS1L	Coding	-0.219	-0.383	148	-14.2	0	0.006	0	0	0	0	0	0	1	0	0	0	0	0	4900
NM_006620	HBS1L	Coding	-0.219	-0.383	148	-14.2	0	0.006	0	0	0	0	0	0	1	0	0	0	0	0	4900
NM_0013182	QPRT	Coding	-0.218	-0.261	146	-14.75	0	0.006	0	0	0	0	0	0	1	0	0	0	0	0	1289
NM_0013182	QPRT	Coding	-0.218	-0.261	146	-14.75	0	0.006	0	0	0	0	0	0	1	0	0	0	0	0	1289
NM_014298	QPRT	Coding	-0.218	-0.261	146	-14.75	0	0.006	0	0	0	0	0	0	1	0	0	0	0	0	1289
NM_018453	EAPP	Coding	-0.218	-0.277	150	-10.9	0	0.11	0	0	0	0	0	0	0	1	0	0	0	0	381
NM_144657	HDX	Coding	-0.218	-0.319	159	-22.16	0	0.006	0	0	0	0	0	0	1	0	0	0	0	0	4015
NM_0011774	HDX	Coding	-0.218	-0.319	159	-22.16	0	0.006	0	0	0	0	0	0	1	0	0	0	0	0	4015

NM_0011774	HDX	Coding	-0.218	-0.319	159	-22.16	0	0.006	0	0	0	0	0	0	1	0	0	0	0	0	4015
NM_0011026	OTUD4	Coding	-0.218	-0.356	150	-15.13	0	0.006	0	0	0	0	0	0	1	0	0	0	0	0	3780
NM_0010252	DUT	Coding	-0.218	-0.365	150	-14.59	0	0.006	0	0	0	0	0	0	1	0	0	0	0	0	1295
NM_0010252	DUT	Coding	-0.218	-0.365	150	-14.59	0	0.006	0	0	0	0	0	0	1	0	0	0	0	0	1295
NM_001948	DUT	Coding	-0.218	-0.365	150	-14.59	0	0.006	0	0	0	0	0	0	1	0	0	0	0	0	1295
NM_153015	TMEM74	Coding	-0.217	-0.386	147	-14.59	0	0.006	0	0	0	0	0	0	1	0	0	0	0	0	5205
NM_0011776	HABP2	Coding	-0.217	-0.226	142	-16.83	0	0.006	0	0	0	0	0	0	1	0	0	0	0	0	1232
NM_004132	HABP2	Coding	-0.217	-0.226	142	-16.83	0	0.006	0	0	0	0	0	0	1	0	0	0	0	0	1232
NM_032547	SCOC	Coding	-0.217	-0.317	152	-13.75	0	0.11	0	0	0	0	0	0	0	1	0	0	0	0	1452
NM_0011534	SCOC	Coding	-0.217	-0.317	152	-13.75	0	0.11	0	0	0	0	0	0	0	1	0	0	0	0	1452
NM_0011536	SCOC	Coding	-0.217	-0.317	152	-13.75	0	0.11	0	0	0	0	0	0	0	1	0	0	0	0	1452
NM_0011535	SCOC	Coding	-0.217	-0.317	152	-13.75	0	0.11	0	0	0	0	0	0	0	1	0	0	0	0	1452
NM_0011536	SCOC	Coding	-0.217	-0.317	152	-13.75	0	0.11	0	0	0	0	0	0	0	1	0	0	0	0	1452
NM_0011534	SCOC	Coding	-0.217	-0.317	152	-13.75	0	0.11	0	0	0	0	0	0	0	1	0	0	0	0	1452
NM_0011535	SCOC	Coding	-0.217	-0.317	152	-13.75	0	0.11	0	0	0	0	0	0	0	1	0	0	0	0	1452
NM_0011536	SCOC	Coding	-0.217	-0.317	152	-13.75	0	0.11	0	0	0	0	0	0	0	1	0	0	0	0	1452
NM_0012584	SLC4A8	Coding	-0.217	-0.26	142	-14.33	0	0.006	0	0	0	0	0	0	1	0	0	0	0	0	8325
NM_0010399	SLC4A8	Coding	-0.217	-0.26	142	-14.33	0	0.006	0	0	0	0	0	0	1	0	0	0	0	0	8325
NM_0010996	GXYLT1	Coding	-0.216	-0.313	149	-13.74	0	0.11	0	0	0	0	0	0	0	1	0	0	0	0	5940
NM_173601	GXYLT1	Coding	-0.216	-0.313	149	-13.74	0	0.11	0	0	0	0	0	0	0	1	0	0	0	0	5940
NM_0012771	EFS	Coding	-0.216	-0.247	146	-15.35	0	0.006	0	0	0	0	0	0	1	0	0	0	0	0	826
NM_005864	EFS	Coding	-0.216	-0.247	146	-15.35	0	0.006	0	0	0	0	0	0	1	0	0	0	0	0	826
NM_032459	EFS	Coding	-0.216	-0.247	146	-15.35	0	0.006	0	0	0	0	0	0	1	0	0	0	0	0	826
NM_172225	DMBX1	Coding	-0.215	-0.252	151	-15.61	0	0.006	0	0	0	0	0	0	1	0	0	0	0	0	1720
NM_147192	DMBX1	Coding	-0.215	-0.252	151	-15.61	0	0.006	0	0	0	0	0	0	1	0	0	0	0	0	1720
NM_024786	ZDHHC11	Coding	-0.215	-0.233	150	-15.88	0	0.006	0	0	0	0	0	0	1	0	0	0	0	0	983
NM_006652	SPINT3	Coding	-0.214	-0.284	181	-28.18	0	0.11	0	0	0	0	0	0	0	1	0	0	0	0	190
NM_0012782	LMCD1	Coding	-0.214	-0.26	154	-15.74	0	0.11	0	0	0	0	0	0	0	1	0	0	0	0	1381
NM_0011453	PLIN1	Coding	-0.213	-0.275	148	-12.44	0	0.11	0	0	0	0	0	0	0	1	0	0	0	0	1214
NM_002666	PLIN1	Coding	-0.213	-0.275	148	-12.44	0	0.11	0	0	0	0	0	0	0	1	0	0	0	0	1214
NM_152400	C4orf32	Coding	-0.213	-0.354	155	-17.1	0	0.006	0	0	0	0	0	0	1	0	0	0	0	0	2143
NM_0011682	SLFNL1	Coding	-0.213	-0.258	142	-12.94	0	0.006	0	0	0	0	0	0	1	0	0	0	0	0	509
NM_0013008	SLFNL1	Coding	-0.213	-0.258	142	-12.94	0	0.006	0	0	0	0	0	0	1	0	0	0	0	0	509
NM_144990	SLFNL1	Coding	-0.213	-0.258	142	-12.94	0	0.006	0	0	0	0	0	0	1	0	0	0	0	0	509
NM_0012000	NPL	Coding	-0.213	-0.279	146	-11.81	0	0.11	0	0	0	0	0	0	0	1	0	0	0	0	705
NM_0012000	NPL	Coding	-0.213	-0.279	146	-11.81	0	0.11	0	0	0	0	0	0	0	1	0	0	0	0	705
NM_0013079	ARID3B	Coding	-0.213	-0.258	144	-13.92	0	0.006	0	0	0	0	0	0	1	0	0	0	0	0	2357
NM_006465	ARID3B	Coding	-0.213	-0.258	144	-13.92	0	0.006	0	0	0	0	0	0	1	0	0	0	0	0	2357
NM_0012025	NIPSNAP1	Coding	-0.213	-0.263	158	-13.45	0	0.006	0	0	0	0	0	0	1	0	0	0	0	0	1126
NM_003634	NIPSNAP1	Coding	-0.213	-0.263	158	-13.45	0	0.006	0	0	0	0	0	0	1	0	0	0	0	0	1126
NM_031244	SIRT5	Coding	-0.212	-0.29	148	-14.3	0	0.006	0	0	0	0	0	0	1	0	0	0	0	0	1220
NM_032126	TEX35	Coding	-0.212	-0.262	156	-16.71	0	0.11	0	0	0	0	0	0	0	1	0	0	0	0	214
NM_0013010	ZBTB44	Coding	-0.211	-0.347	162	-22.22	0	0.006	0	0	0	0	0	0	1	0	0	0	0	0	5038
NM_025153	ATP10B	Coding	-0.211	-0.298	163	-20.09	0	0.006	0	0	0	0	0	0	1	0	0	0	0	0	2333
NM_0012000	NPL	Coding	-0.211	-0.278	146	-11.81	0	0.11	0	0	0	0	0	0	0	1	0	0	0	0	1661
NM_0012000	NPL	Coding	-0.211	-0.278	146	-11.81	0	0.11	0	0	0	0	0	0	0	1	0	0	0	0	1476
NM_030769	NPL	Coding	-0.211	-0.278	146	-11.81	0	0.11	0	0	0	0	0	0	0	1	0	0	0	0	1476
NM_0011436	ZBED5	Coding	-0.211	-0.258	152	-14.42	0	0.11	0	0	0	0	0	0	0	1	0	0	0	0	160
NM_021211	ZBED5	Coding	-0.211	-0.258	152	-14.42	0	0.11	0	0	0	0	0	0	0	1	0	0	0	0	160
NM_144659	TCP10L	Coding	-0.211	-0.229	160	-19.85	0	0.006	0	0	0	0	0	0	1	0	0	0	0	0	1933
NM_0011708	LARP4	Coding	-0.21	-0.261	145	-10.53	0	0.11	0	0	0	0	0	0	0	1	0	0	0	0	4141
NM_0011708	LARP4	Coding	-0.21	-0.261	145	-10.53	0	0.11	0	0	0	0	0	0	0	1	0	0	0	0	4141
NM_052879	LARP4	Coding	-0.21	-0.261	145	-10.53	0	0.11	0	0	0	0	0	0	0	1	0	0	0	0	4141
NM_199188	LARP4	Coding	-0.21	-0.261	145	-10.53	0	0.11	0	0	0	0	0	0	0	1	0	0	0	0	4141
NM_199190	LARP4	Coding	-0.21	-0.261	145	-10.53	0	0.11	0	0	0	0	0	0	0	1	0	0	0	0	4141
NM_0012785	CORIN	Coding	-0.21	-0.282	156	-14.77	0	0.11	0	0	0	0	0	0	0	1	0	0	0	0	1723
NM_006587	CORIN	Coding	-0.21	-0.282	156	-14.77	0	0.11	0	0	0	0	0	0	0	1	0	0	0	0	1723
NM_0011450	SCRN2	Coding	-0.209	-0.187	144	-16.03	0	0.006	0	0	0	0	0	0	1	0	0	0	0	0	571
NM_025268	TMEM121	Coding	-0.209	-0.241	158	-17.26	0	0.006	0	0	0	0	0	0	1	0	0	0	0	0	408
NM_0011301	MYO1B	Coding	-0.209	-0.267	155	-12.95	0	0.11	0	0	0	0	0	0	0	1	0	0	0	0	1429

NM_012223	MYO1B	Coding	-0.209	-0.267	155	-12.95	0	0.11	0	0	0	0	0	0	0	1	0	0	0	0	1429
NM_0011618	MYO1B	Coding	-0.209	-0.267	155	-12.95	0	0.11	0	0	0	0	0	0	0	1	0	0	0	0	1429
NM_001110	ADAM10	Coding	-0.209	-0.264	151	-12.98	0	0.11	0	0	0	0	0	0	0	1	0	0	0	0	2343
NM_0013205	ADAM10	Coding	-0.209	-0.264	151	-12.98	0	0.11	0	0	0	0	0	0	0	1	0	0	0	0	2343
NM_0012708	MEAF6	Coding	-0.208	-0.279	149	-13.43	0	0.11	0	0	0	0	0	0	0	1	0	0	0	0	3809
NM_0013205	ADD3	Coding	-0.207	-0.257	147	-15.96	0	0.11	0	0	0	0	0	0	0	1	0	0	0	0	1947
NM_0013205	ADD3	Coding	-0.207	-0.257	147	-15.96	0	0.11	0	0	0	0	0	0	0	1	0	0	0	0	1947
NM_019903	ADD3	Coding	-0.207	-0.257	147	-15.96	0	0.11	0	0	0	0	0	0	0	1	0	0	0	0	1947
NM_001121	ADD3	Coding	-0.207	-0.257	147	-15.96	0	0.11	0	0	0	0	0	0	0	1	0	0	0	0	1947
NM_0013205	ADD3	Coding	-0.207	-0.257	147	-15.96	0	0.11	0	0	0	0	0	0	0	1	0	0	0	0	1947
NM_016824	ADD3	Coding	-0.207	-0.257	147	-15.96	0	0.11	0	0	0	0	0	0	0	1	0	0	0	0	1947
NM_0013205	ADD3	Coding	-0.207	-0.257	147	-15.96	0	0.11	0	0	0	0	0	0	0	1	0	0	0	0	1947
NM_000088	COL1A1	Coding	-0.207	-0.259	153	-13.55	0	0.11	0	0	0	0	0	0	0	1	0	0	0	0	1406
NM_018356	C5orf22	Coding	-0.206	-0.268	147	-14.94	0	0.11	0	0	0	0	0	0	0	1	0	0	0	0	2156
NM_020393	PGLYRP4	Coding	-0.206	-0.25	149	-15.5	0	0.11	0	0	0	0	0	0	0	1	0	0	0	0	646
NM_0013189	EAPP	Coding	-0.206	-0.269	150	-10.9	0	0.11	0	0	0	0	0	0	0	1	0	0	0	0	701
NM_001158	AOC2	Coding	-0.206	-0.255	146	-12.98	0	0.11	0	0	0	0	0	0	0	1	0	0	0	0	359
NM_009590	AOC2	Coding	-0.206	-0.255	146	-12.98	0	0.11	0	0	0	0	0	0	0	1	0	0	0	0	359
NM_152860	SP7	Coding	-0.206	-0.208	146	-13.38	0	0.006	0	0	0	0	0	0	1	0	0	0	0	0	1570
NM_0011734	SP7	Coding	-0.206	-0.208	146	-13.38	0	0.006	0	0	0	0	0	0	1	0	0	0	0	0	1570
NM_0013008	SP7	Coding	-0.206	-0.208	146	-13.38	0	0.006	0	0	0	0	0	0	1	0	0	0	0	0	1570
NM_152601	ZNF709	Coding	-0.205	-0.281	152	-19.17	0	0.11	0	0	0	0	0	0	0	1	0	0	0	0	2812
NM_0013217	ZNF471	Coding	-0.205	-0.222	149	-21.13	0	0.006	0	0	0	0	0	0	1	0	0	0	0	0	4266
NM_020813	ZNF471	Coding	-0.205	-0.222	149	-21.13	0	0.006	0	0	0	0	0	0	1	0	0	0	0	0	4266
NM_0010173	SUGP2	Coding	-0.205	-0.281	160	-18.19	0	0.006	0	0	0	0	0	0	1	0	0	0	0	0	2854
NM_0013216	SUGP2	Coding	-0.205	-0.281	160	-18.19	0	0.006	0	0	0	0	0	0	1	0	0	0	0	0	2854
NM_0012561	S100PBP	Coding	-0.205	-0.283	160	-19.24	0	0.11	0	0	0	0	0	0	0	1	0	0	0	0	2841
NM_022753	S100PBP	Coding	-0.205	-0.283	160	-19.24	0	0.11	0	0	0	0	0	0	0	1	0	0	0	0	2841
NM_017998	C9orf40	Coding	-0.204	-0.244	148	-15.59	0	0.11	0	0	0	0	0	0	0	1	0	0	0	0	1465
NM_0011989	CCDC169-SOHLH2	Coding	-0.204	-0.255	154	-14.66	0	0.11	0	0	0	0	0	0	0	1	0	0	0	0	826
NM_017826	SOHLH2	Coding	-0.204	-0.255	154	-14.66	0	0.11	0	0	0	0	0	0	0	1	0	0	0	0	826
NM_0011992	LRBA	Coding	-0.204	-0.261	146	-12.35	0	0.11	0	0	0	0	0	0	0	1	0	0	0	0	1063
NM_006726	LRBA	Coding	-0.204	-0.261	146	-12.35	0	0.11	0	0	0	0	0	0	0	1	0	0	0	0	1063
NM_004642	CDK2AP1	Coding	-0.204	-0.273	159	-20.93	0	0.11	0	0	0	0	0	0	0	1	0	0	0	0	766
NM_0012704	CDK2AP1	Coding	-0.204	-0.273	159	-20.93	0	0.11	0	0	0	0	0	0	0	1	0	0	0	0	766
NM_0012704	CDK2AP1	Coding	-0.204	-0.273	159	-20.93	0	0.11	0	0	0	0	0	0	0	1	0	0	0	0	766
NM_0011904	CTH	Coding	-0.203	-0.26	142	-12.35	0	0.11	0	0	0	0	0	0	0	1	0	0	0	0	724
NM_001902	CTH	Coding	-0.203	-0.26	142	-12.35	0	0.11	0	0	0	0	0	0	0	1	0	0	0	0	724
NM_153742	CTH	Coding	-0.203	-0.26	142	-12.35	0	0.11	0	0	0	0	0	0	0	1	0	0	0	0	724
NM_0012860	TAF1	Coding	-0.203	-0.335	148	-16.02	0	0.006	0	0	0	0	0	0	1	0	0	0	0	0	1959
NM_004606	TAF1	Coding	-0.203	-0.335	148	-16.02	0	0.006	0	0	0	0	0	0	1	0	0	0	0	0	1959
NM_138923	TAF1	Coding	-0.203	-0.335	148	-16.02	0	0.006	0	0	0	0	0	0	1	0	0	0	0	0	1959
NM_017896	GID8	Coding	-0.203	-0.363	147	-16.33	0	0.006	0	0	0	0	0	0	1	0	0	0	0	0	3563
NM_0013042	LDLRAD3	Coding	-0.203	-0.28	148	-14.4	0	0.11	0	0	0	0	0	0	0	1	0	0	0	0	2739
NM_0013042	LDLRAD3	Coding	-0.203	-0.28	148	-14.4	0	0.11	0	0	0	0	0	0	0	1	0	0	0	0	2739
NM_174902	LDLRAD3	Coding	-0.203	-0.28	148	-14.4	0	0.11	0	0	0	0	0	0	0	1	0	0	0	0	2739
NM_000761	CYP1A2	Coding	-0.202	-0.217	164	-26.25	0	0.006	0	0	0	0	0	0	1	0	0	0	0	0	1512
NM_002834	PTPN11	Coding	-0.202	-0.253	140	-9.89	0	0.11	0	0	0	0	0	0	0	1	0	0	0	0	4121
NM_000769	CYP2C19	Coding	-0.202	-0.247	142	-14.02	0	0.006	0	0	0	0	0	0	1	0	0	0	0	0	291
NM_003437	ZNF136	Coding	-0.202	-0.283	144	-14.01	0	0.006	0	0	0	0	0	0	1	0	0	0	0	0	1248
NM_033050	SUCNR1	Coding	-0.202	-0.321	148	-16.44	0	0.006	0	0	0	0	0	0	1	0	0	0	0	0	3071
NM_024516	PAGR1	Coding	-0.201	-0.286	166	-20.78	0	0.11	0	0	0	0	0	0	0	1	0	0	0	0	2741
NM_0011644	GOLGA6C	Coding	-0.201	-0.272	165	-23.93	0	0.11	0	0	0	0	0	0	0	1	0	0	0	0	3256
NM_004946	DOCK2	Coding	-0.2	-0.248	142	-10.03	0	0.11	0	0	0	0	0	0	0	1	0	0	0	0	524
NM_014879	P2RY14	Coding	-0.2	-0.239	140	-9.57	0	0.11	0	0	0	0	0	0	0	1	0	0	0	0	1183
NM_0010814	P2RY14	Coding	-0.2	-0.239	140	-9.57	0	0.11	0	0	0	0	0	0	0	1	0	0	0	0	1183
